# DrugReSC: targeting disease-critical cell subpopulations with single-cell transcriptomic data for drug repurposing in cancer

**DOI:** 10.1093/bib/bbae490

**Published:** 2024-09-30

**Authors:** Chonghui Liu, Yan Zhang, Yingjian Liang, Tianjiao Zhang, Guohua Wang

**Affiliations:** College of Life Science, Northeast Forestry University, 26 Hexing Road, Xiangfang District, Harbin 150040, China; College of Computer and Control Engineering, Northeast Forestry University, 26 Hexing Road, Xiangfang District, Harbin 150040, China; Kunming Institute of Zoology, Chinese Academy of Sciences, 17 Longxin Road, Panlong District, Kunming 650201, Yunnan, China; University of Chinese Academy of Sciences, 1 Yanxi Lake East Road, Huairou District, Beijing 100049, China; Department of General Surgery, the First Affiliated Hospital of Harbin Medical University, 23 Youzheng Street, Nangang District, Harbin 150007, China; College of Computer and Control Engineering, Northeast Forestry University, 26 Hexing Road, Xiangfang District, Harbin 150040, China; College of Computer and Control Engineering, Northeast Forestry University, 26 Hexing Road, Xiangfang District, Harbin 150040, China

**Keywords:** heterogeneity, drug discovery, melanoma, lung cancer, machine learning, gene signature

## Abstract

The field of computational drug repurposing aims to uncover novel therapeutic applications for existing drugs through high-throughput data analysis. However, there is a scarcity of drug repurposing methods leveraging the cellular-level information provided by single-cell RNA sequencing data. To address this need, we propose DrugReSC, an innovative approach to drug repurposing utilizing single-cell RNA sequencing data, intending to target specific cell subpopulations critical to disease pathology. DrugReSC constructs a drug-by-cell matrix representing the transcriptional relationships between individual cells and drugs and utilizes permutation-based methods to assess drug contributions to cellular phenotypic changes. We demonstrate DrugReSC’s superior performance compared to existing drug repurposing methods based on bulk or single-cell RNA sequencing data across multiple cancer case studies. In summary, DrugReSC offers a novel perspective on the utilization of single-cell sequencing data in drug repurposing methods, contributing to the advancement of precision medicine for cancer.

## Introduction

Intra-tumor heterogeneity presents a significant challenge to achieving effective cancer treatment outcomes [1]. While tumor heterogeneity is often attributed to genetic evolution, the specific underlying mechanisms remain incompletely understood [2]. Recently, single-cell RNA-sequencing (scRNA-seq) has emerged as a promising tool for investigating the intra-tumor heterogeneity across various cancer types [3–5]. Despite providing rich cellular-level information, current single-cell datasets lack direct associations between cell clusters and specific diseases, such as cancer. Numerous studies have revealed that disease progression is frequently driven by changes in only a small subset of key cells [6–8]. However, there is currently limited research focusing on key cell populations closely associated with cancer. Therefore, comprehending the expression signature of critical cells within tumor tissues and identifying potential drugs to target these specific cell subpopulations are crucial aspects of cancer drug therapy.

Several recent studies have begun to address these challenges. For instance, single-cell RNA sequencing has been used to identify multiple cancer cell subpopulations within tumors, demonstrating differential therapeutic sensitivity and aiding in the prediction and validation of small molecules that target drug-tolerant cell populations [9]. Another study developed a single-cell atlas for breast cancer cell lines, which enabled the prediction of drug responses based on single-cell profiles [10]. Additionally, single-cell ‘mass cytometry’ has provided system-wide views of immune signaling in hematopoiesis, highlighting the potential for single-cell analyses to inform drug action and disease mechanisms [11].

Drug repurposing, also known as drug repositioning, involves repurposing existing compounds approved for specific disorders for new clinical indications [12]. This strategy enables the expedited clinical deployment of drugs at a reduced cost compared to the traditional de novo drug discovery process [13]. Despite its potential, there has been limited development of drug repurposing methods designed to leverage the detailed cellular-level information provided by single-cell RNA sequencing data.

ASGARD is one method developed to leverage single-cell data for drug prediction. It integrates drug prediction outcomes across multiple selected cell clusters for each patient, establishing a comprehensive drug scoring system [14]. However, ASGARD requires single-cell data from both diseased patients and normal controls, which can be limiting in practical scenarios where only patient data is available.

Another approach, Beyondcell, identifies tumor cell subpopulations with distinct drug responses by calculating enrichment scores across drug signatures, thereby delineating therapeutic clusters within cellular populations [15]. Similarly, scDEAL employs a domain-adaptive neural network within a deep transfer learning framework to predict cancer drug response at the single-cell level by integrating large-scale bulk cell-line data [16]. Despite their advancements, both Beyondcell and scDEAL overlook the role of crucial disease-relevant cells that may be dispersed across different cell clusters, leading to potential inaccuracies when drug predictions are based solely on cell clusters.

Hence, we introduce a Drug  Re purposing approach based on disease-critical cell subpopulations identified from Single-Cell RNA sequencing data (DrugReSC) to overcome the limitations above. DrugReSC initially employs our previously developed PACSI method to identify disease-associated cells in single-cell sequencing data [17]. Subsequently, it constructs a drug-by-cell matrix to evaluate the relationship between each drug and each cell. Ultimately, DrugReSC obtains a ranked list of candidate drugs through permutation-based methods. We evaluate DrugReSC across multiple cancer cases, including melanoma, non-small cell lung cancer, and renal cell carcinoma. The results demonstrate that DrugReSC outperforms existing drug repurposing methods based on bulk and single-cell RNA sequencing data. Our research demonstrates that DrugReSC is a well-performing drug repurposing tool based on single-cell data, providing new insights into precision medicine for diseases.

## Methods

### scRNA data and preprocessing

We acquired several scRNA datasets from the Gene Expression Omnibus (GEO) database. The scRNA data obtained from melanoma patients were sourced from ‘GSE189889.’ We excluded cells from three lymph node metastasis samples and retained only the cells originating from primary tumors in the dataset. We also obtained scRNA-seq data from tumor tissues of NSCLC patients from the GEO dataset with accession number GSE140819. Furthermore, the scRNA-seq data of renal cell carcinoma patients were obtained from GEO with accession number GSE152938. We filtered out cells from samples of papillary renal cell carcinoma and chromophobe renal cell carcinoma, retaining only cells from two clear cell renal cell carcinoma tissue samples and one normal kidney tissue sample. In addition, we downloaded scRNA-seq data from normal control skin samples of 10 healthy volunteers from the GEO dataset with accession number GSE183047. The scRNA-seq data of normal lung tissue were obtained from GEO with accession number GSE122960. Before running DrugReSC, the read counts for individual genes in the single-cell datasets were transformed into transcript per million quantification using the IOBR package [18], followed by log2 transformation.

### Bulk RNA sequencing data and preprocessing

We obtained melanoma bulk gene expression data, quantified as Fragments Per Kilobase Million, along with corresponding phenotype information, from the TCGA-SKCM dataset through the UCSC Xena platform [19]. The dataset comprised a total of 472 samples, excluding one ‘Additional Metastatic’ and one ‘Solid Tissue Normal’ sample, resulting in 367 metastatic samples and 103 primary tumor samples. We focused on metastasis as the phenotype of interest and utilized DrugReSC to identify cells associated with melanoma metastasis. Likewise, we retrieved bulk gene expression data for NSCLC from the TCGA-LUAD dataset and renal cell carcinoma (RCC) from the TCGA-KIRC dataset. The NSCLC dataset comprised 585 samples, with 526 tumor and 59 normal tissue samples. In the RCC dataset, we excluded one ‘Additional - New Primary’ sample, leaving 606 samples, including 534 tumor and 72 normal tissue samples. Samples originating from tumor tissues were considered the phenotype of interest. During the conversion of Ensembl IDs to gene symbols for bulk gene expression data, the mean expression values were utilized for genes sharing the same gene symbol.

### Drug data

All positive drugs for the indications were obtained from the DrugBank database [20]. We obtained a list of 17 drugs associated with melanoma by querying the DrugBank database with the term ‘Metastatic Melanoma’ (Supplementary Table 1). Additionally, we identified 28 drugs related to NSCLC by querying ‘Metastatic Non-Small Cell Lung Cancer’ (Supplementary Table 1). We retrieved 10 drugs associated with renal cell carcinoma through a search for ‘Advanced Renal Cell Carcinoma’ (Supplementary Table 1). The drug-perturbed expression profiles were obtained from the LINCS project, comprising assay results derived from primary human cells subjected to chemical perturbations or left untreated [21]. We matched drugs obtained from the DrugBank database with their corresponding drug treatment instances in the LINCS L1000 project, designating them as positive drug instances, each representing a single replicate of a drug treatment condition (e.g., concentration, cell line, duration, etc.). Subsequently, we randomly selected an equal number of drug instances from the corresponding tissues in the LINCS L1000 project as negative drug instances, to account for the absence of publicly available data on drug-disease negative relationships. To address potential randomness in our selection process, we performed 10 samplings for each indication, resulting in 10 lists of negative drug instances. Subsequently, we combined the positive drug instance list with each of the 10 negative drug instance lists, resulting in 10 ground truth lists for each indication (Supplementary Tables 2–4). We then assessed the performance of drug repurposing methods for each indication using these 10 ground truth lists.

### Description of DrugReSC

#### Identification of cancer-associated cells

PACSI, a method previously developed by our team, integrated information from both single-cell and bulk RNA sequencing to identify cells associated with disease phenotypes [17]. DrugReSC employed the PACSI approach as a first step to identify cells associated with the disease using both disease-specific scRNA-seq data and bulk gene expression data. The phenotype of the identified disease-associated cells was set to 1, while the phenotype of other cells was set to 0.

#### Construction of drug signatures

The drug-induced gene expression profiles generated by the LINCS L1000 project in skin, lung, and kidney tissues were extracted separately from the iLINCS database [22]. The drug-perturbed expression profiles comprised assay results obtained from primary human cells treated with or without chemical perturbations. We retained profiles measured at 10 μM of drug treatment, as this concentration was the most common across all experiments. For each drug instance, a differential expression analysis was conducted to obtain two vectors: the vector of log-scale differential expressions between the drug-treated samples and control samples $\boldsymbol{c}=\left({c}_1,\dots, {c}_N\right)$, and the vector of associated *P* values $\textbf{p}=\left({p}_1,\dots, {p}_N\right)$, where N represents the number of genes. Genes with log-scale differential expressions greater than 0 and associated *P* values less than 0.01 were extracted to form the up-signature for each drug instance. Similarly, genes with log-scale differential expressions less than 0 and associated *P* values less than 0.01 were extracted to compose the down-signature for each drug instance. In this manner, an up-signature and a down-signature were created for each drug instance in ground truth lists.

#### Drug-by-cell matrix transformation

To establish the relationship between each drug instance and each cell, we defined a drug-to-cell (D2C) score for each drug instance $d$ in a cell $c$, calculated as follows:


(1) \begin{equation*} D2C\ \mathrm{score}=E{S}_{up}-E{S}_{down}, \end{equation*}

Where $E{S}_{up}$ and $E{S}_{down}$ represented the enrichment scores of the up-signature and down-signature of drug $d$ in cell $c$, respectively. The enrichment score $ES$ for a gene signature $G$ in each cell $c$ was calculated using the single-sample gene set enrichment analysis (ssGSEA) method [23]. Specifically, DrugReSC first ranked the $N$ genes in cell $c$ by their absolute expression values to form the list $L=\left\{{r}_1,{r}_2,\dots, {r}_N\right\}$. The list was subsequently organized in descending order, ranging from the highest rank $N$ to the lowest rank 1. Then, for a gene signature $G$ of size $NG$, DrugReSC evaluated the fraction of genes in $G$ (‘hits’) weighted by their ranks and the fraction of genes not in $G$ (‘misses’) present up to a given position $i$ in $L$: 


(2) \begin{equation*} {\displaystyle \begin{array}{c}{P}_{\mathrm{hit}}\left(G,i\right)=\sum\limits_{\begin{array}{c}\mathrm{gene}j\in G\\{}j\le i\end{array}}\frac{{\left|{r}_j\right|}^{\alpha }}{\sum_{\mathrm{gene}j\in G}{\left|{r}_j\right|}^{\alpha}\kern0.1em },\end{array}} \end{equation*}

(3) \begin{equation*} {\displaystyle \begin{array}{c}{P}_{\mathrm{miss}}\left(G,i\right)=\sum\limits_{\begin{array}{c} genej\notin G\\{}j\le i\end{array}}\frac{1}{\left(N- NG\right)}.\end{array}} \end{equation*}

The $ES$ was the maximum deviation from zero of ${P}_{hit}-{P}_{miss}$. The exponent of this quantity $\alpha$ was set to 0.25.

Through the described computational procedure, we obtained the $D2C$ scores for each drug instance $d$ within a given cell $c$, enabling the transformation of a gene-by-cell matrix into a drug-by-cell matrix. This conversion effectively represented the associations between drugs and cells.

#### Calculation of the contribution of drugs

To determine the contribution of each drug instance to cell phenotype changes, we first established a random forest model on the drug-by-cell matrix with cell phenotype (disease-related or disease-unrelated) as the dependent variable. The parameter for the number of trees in the random forest model was set to 100, and the number of candidate variables randomly sampled at each split was set to the value corresponding to the minimum error rate (Supplementary Figs. 1–3). The importance of each independent variable (drug instance) in the model represented its contribution to cell phenotype changes. To assess the importance of each independent variable, we permuted the values of independent variable ${\mathrm{X}}_d$ in the out-of-bag observations and reintroduced these observations into the tree [24]. The out-of-bag sample, comprising observations not utilized for constructing the current tree, served both to estimate prediction error and to evaluate variable importance. If the independent variable lacked association with the response, permuting its values has negligible impact on classification, yielding an importance score close to zero. Conversely, if there was an association between the response and the independent variable, permuting its values leads to decreased predictive accuracy. Specifically, the importance score ${Drug\ score}_d$ of independent variable ${\mathrm{X}}_d$ was defined as follows:


(4) \begin{equation*} {\displaystyle \begin{array}{c}{Drug\ score}_d=\frac{1}{\mathrm{ntree}}\sum_{t=1}\limits^{\mathrm{ntree}}\left({C}_{td}-C{P}_{td}\right),\end{array}} \end{equation*}

where $\mathit{\mathsf{ntree}}$ represented the number of trees in the forest, ${C_{td}}$ signified the number of votes for the correct class of tree $t$ when predicting all out-of-bag observations before permuting the values of variable ${X_d}$, and $C{P_{td}}$ indicated the number of votes for the correct class of tree ${t}$ when predicting all out-of-bag observations after randomly permuting the values of variable ${X_d}$. The formula for calculating the number of votes for the correct class $C$ of predicting out-of-bag observations for each tree was as follows:


(5) \begin{equation*} {\displaystyle \begin{array}{c}C=\sum\limits_{k=1}^nI\left({\overset{\hat{\mkern2mu{Y}}}{}}_k={Y}_k\right),\end{array}} \end{equation*}

where $n$ represented the total number of observations, ${Y}_k$ represented the true classes, ${\overset{\hat{Y}}{}}_k$ represented the predicted classes, and $I\left(\bullet \right)$ denoted the indicator function.

To facilitate the comparison of drug scores, DrugReSC additionally offered a standardized drug score, ranging from 0 to 1:


(6) \begin{equation*} {\displaystyle \begin{array}{c} Standardized\ Drug\ Score=\frac{Drug\ score-\mathit{\operatorname{MIN}}\left( Drug\ score\right)}{\mathit{\operatorname{MAX}}\left( Drug\ score\right)-\mathit{\operatorname{MIN}}\left( Drug\ score\right)},\end{array}} \end{equation*}

where $\mathit{\operatorname{MAX}}\left( Drug\ score\right)$ denoted the maximum drug score and $\mathit{\operatorname{MIN}}\left( Drug\ score\right)$ denoted the minimum drug score.

### Alternative strategies for evaluating feature importance

To assess the contribution of each drug to cell phenotype classification within the D2C matrix, we employed several additional established statistical and machine learning methods, namely logistic regression, support vector machine (SVM), analysis of variance, and the Wilcoxon rank-sum test. Each of these methods offered unique advantages in assessing the importance of independent variables.

Logistic Regression: initially, L1 regularization was employed in the logistic regression model to mitigate overfitting. Model training was conducted using 5-fold cross-validation to determine the optimal regularization strength. Subsequently, based on the identified minimum regularization strength, the final logistic regression model was constructed. The coefficients associated with each feature in the model’s output represented the importance scores of the drug instances.

Support Vector Machine (SVM): the SVM model was constructed using the Radial Basis Function Kernel as the kernel function. Additionally, we employed the one-dimensional sensitivity analysis method to calculate the importance of drug instances [25, 26].

Analysis of Variance (ANOVA): we utilized analysis of variance to compute the F-statistics for each drug instance in the D2C matrix, representing the importance of drug instances. Before conducting the ANOVA, the D2C scores of each drug across disease-associated and disease-unassociated cell groups were assessed using the Bartlett’s test. For drug instances exhibiting homogeneity of variance, we performed one-way ANOVA, while for those with heterogeneous variances, we applied the Welch method to calculate the importance of drug instances [27].

Wilcoxon rank-sum test: the significance of differences in D2C scores of each drug instance between disease-associated and disease-unassociated cell groups was evaluated using the Wilcoxon rank-sum test. A smaller p-value indicated a more significant difference, suggesting a higher importance of the drug instance.

XGBoost: eXtreme Gradient Boosting (XGBoost) employed an ensemble approach, leveraging a boosting algorithm to amalgamate multiple weak learners, resulting in a more powerful predictive model [28]. We utilized the relative importance of XGBoost Gain to determine the relative contribution of each drug instance to our predicted cell phenotypes. The maximum boosting iterations of the XGBoost model were set to 500, while the remaining parameters were kept at their default values.

### Comparison of DrugReSC with existing drug repurposing approaches

To compare DrugReSC with other established drug repurposing methods based on disease bulk transcriptome data, we evaluated the performance of each method using the same disease expression profile data and ground truth drug lists. For iLINCS [22], we first employed the R package limma [29] to identify significantly upregulated (*P* value <0.01 and log_2_ fold-change >1) and downregulated (*P* value <0.01 and log_2_ fold-change < −1) genes in the bulk expression profile data of the disease samples. The disease signatures, presented as lists of upregulated and downregulated genes, were submitted to web-based iLINCS tools to query drug instances that ‘reverse’ the disease signature [22]. For DrInsight [30], we initially ranked the vector of log-scale differential expressions between the drug-treated samples and control samples for each drug instance to obtain a drug rank matrix for each disease. Subsequently, we utilized both the drug rank matrix and the log_2_ fold-change values of all genes obtained from the differential expression analysis between disease and control tissue samples as inputs to the DrInsight method for connectivity matching, thereby obtaining the significance level (*P* value) of drug instances.

ASGARD was a method designed to predict candidate drugs based on multiple diseased cell clusters derived from single-cell sequencing data of each patient. The utilization of the ASGARD method followed the guidelines provided by its authors. Additionally, it was worth noting that the ASGARD method required input data from both disease tissues and normal control tissues. Since the NSCLC and melanoma cases only contained single-cell data sourced from tumor tissues, when comparing DrugReSC with ASGARD, the NSCLC and melanoma cases were supplemented with single-cell data from corresponding normal control tissues along with tumor tissue data.

### Alternative methods for the identification of cancer-associated cells

Identifying disease-associated cells was a crucial aspect of the DrugReSC framework. In addition to the PACSI method, we also tested the performance of DrugReSC when using the Scissor [31] and DEGAS [32] methods for the identification of cancer-associated cells within the framework. For Scissor, we utilized its default parameters during execution, designating cells classified as Scissor positive and Scissor negative in the results as disease-associated cells identified by Scissor. For DEGAS, we followed the tutorial provided by the authors. In the results, cells exhibiting a higher association score with the disease phenotype of interest compared to the control phenotype were selected as disease-associated cells identified by DEGAS.

### Evaluation metrics

To assess the predictive performance of DrugReSC, we employed various evaluation metrics. These metrics offer diverse perspectives and collectively provide a comprehensive evaluation of prediction performance across various methodologies.

AUROC: the AUROC score quantifies the area under the receiver operating characteristic curve, which illustrates the trade-off between true positive rate and false positive rate across different thresholds. Utilizing the ‘roc’ function within the pROC package, we conducted AUROC analyses to assess the predictive performance.

AUPR: the AUPR score represents the area under the precision-recall curve, which demonstrates the trade-off between precision and recall. Higher AUPR scores signify superior performance characterized by both high recall and precision.

F1-score: the F1-score provides a harmonic mean of precision and recall, providing a balanced measure of their performance. It reaches its maximum value of 1 when both precision and recall are optimal, and its minimum value of 0 when either precision or recall is at its lowest. The formula for calculating the F1-score is given by:


(6) \begin{equation*} {\displaystyle \begin{array}{c}F1- score=\frac{Ture\ positive}{Ture\ positive\ +\ 0.5\ast \left( Ture\ positive\ +\ False\ positive\right)}.\end{array}} \end{equation*}

Accuracy: accuracy quantifies the proportion of correctly predicted drugs among all drugs evaluated, offering a comprehensive measure of prediction effectiveness.


(7) \begin{equation*} {\displaystyle \begin{array}{c} accuracy=\frac{Ture\ positive\ +\ Ture\ negative}{Ture\ positive\ +\ Ture\ negative\ +\ False\ positive\ +\ False\ negative}.\end{array}} \end{equation*}

### Comparative analysis of published gene sets and functional enrichment analysis

The CancerSEA database provided a curated collection of functional cancer gene sets [33]. To characterize the drug signatures developed in this study, we retrieved 14 gene sets from the CancerSEA database and compared them with our drug signatures. Each drug signature was constructed by merging the up-signature and down-signature of the respective drug instance. The analysis results were performed using the UpSetR package in R [34]. Additionally, Reactome pathway enrichment analyses were conducted using the hypergeometric test, facilitated by the ReactomePA package [35].

### Statistical analysis

Statistical analyses were performed using R (version 4.1.1). Reactome pathway enrichment analysis was performed using the hypergeometric test. We also utilized the Wilcoxon rank-sum test to compare the mean importance scores between positive and negative drug instances. Following on a previous study [36], we employed the RISmed approach [37] to explore supporting evidence regarding the association between specific keywords (e.g., the link between candidate drugs identified by DrugReSC and melanoma) from relevant studies within the PubMed repository. Additionally, we assessed the correlation between drug importance scores and the number of search results on PubMed using Pearson correlation analysis.

## Results

### Overview of the DrugReSC framework

**Figure 1:**
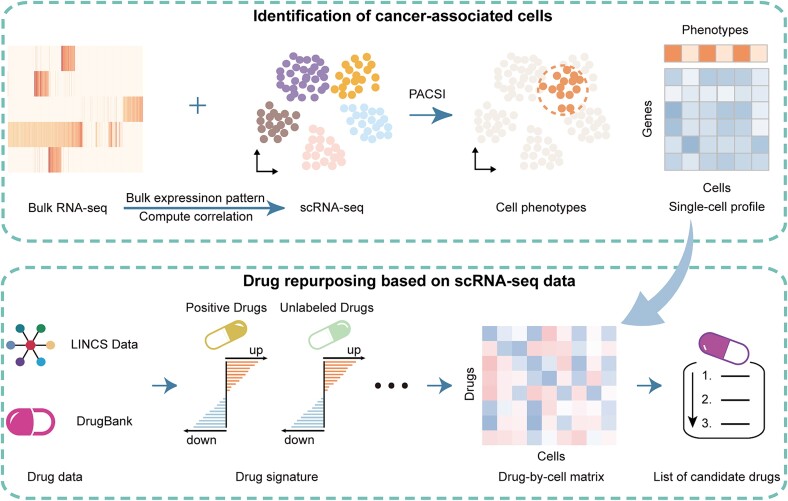
Overview of the DrugReSC method. The framework mainly consists of three steps. (1) DrugReSC initially identifies disease-relevant cells using the PACSI method, based on both single-cell and bulk transcriptomic data (top). Simultaneously, DrugReSC constructs drug signatures from the DrugBank and LINCS databases (bottom left). (2) DrugReSC transforms the single-cell gene-by-cell matrix into a drug-bycell matrix using ssGSEA (bottom center). Each value in the drug-by-cell matrix is defined as a D2C score, representing the degree of reversal of the transcriptional level of a given drug for a specific cell. (3) DrugReSC assesses the contribution of each drug to cellular phenotypic changes using a perturbation-based random forest model and outputs a ranked list of drugs based on their contributions (bottom right).

DrugReSC is a drug repurposing method based on single-cell RNA sequencing data, aiming at targeting disease-relevant cells (Fig. 1). The first step of DrugReSC involves identifying cancer-associated cells using the PACSI method, based on single-cell and bulk gene expression data [17] (Fig. 1, top). Meanwhile, we collect ‘positive drugs’ and ‘unlabeled drugs’ for a particular indication from DrugBank database and obtain the drug signatures from the drug-exposure gene expression of LINCS project [20] (Fig. 1, bottom left). Afterward, DrugReSC transforms the gene-by-cell matrix into a drug-by-cell matrix, utilizing the ssGSEA algorithm based on drug-induced signatures and single-cell expression profiles [23] (Fig. 1, bottom center). Each value in the drug-by-cell matrix is defined as a D2C score, representing the degree of reversal of the transcriptional level of a given drug for a specific cell. A key step in DrugReSC is quantifying the contribution of drugs to cellular phenotype changes in the drug-by-cell matrix, which is measured by permutating the values of the independent variables (drug instance) to evaluate the predictive error of the random forest model. The output candidate drug list from DrugReSC is ranked based on the contributions of the drugs (Fig. 1, bottom right).

### Random forest enables accurate prediction of FDA-approved drugs for indications

Before comparing DrugReSC with other drug repurposing methods, we initially assessed various approaches for quantifying the impact of drug instances on changes in cell phenotypes across three datasets: melanoma [38], non-small cell lung cancer (NSCLC) [39], and RCC [40]. We curated drugs approved by the United States Food and Drug Administration (FDA) from the DrugBank as positive instances and randomly selected an equal number of drugs to serve as negative instances (Methods). Considering the cell phenotypes (disease-related or disease-unrelated) in the drug-by-cell matrix as dependent variables and drug instances as independent variables, we applied several statistical and machine learning methods for feature importance analysis, including the one-way analysis of variance (ANOVA), logistic regression, SVM, Wilcoxon rank-sum test, XGBoost, and random forest (Methods). The performance of drug prediction was systematically evaluated using four metrics: the AUROC, the AUPR, F1-score (F1), and accuracy.

**Figure 2:**
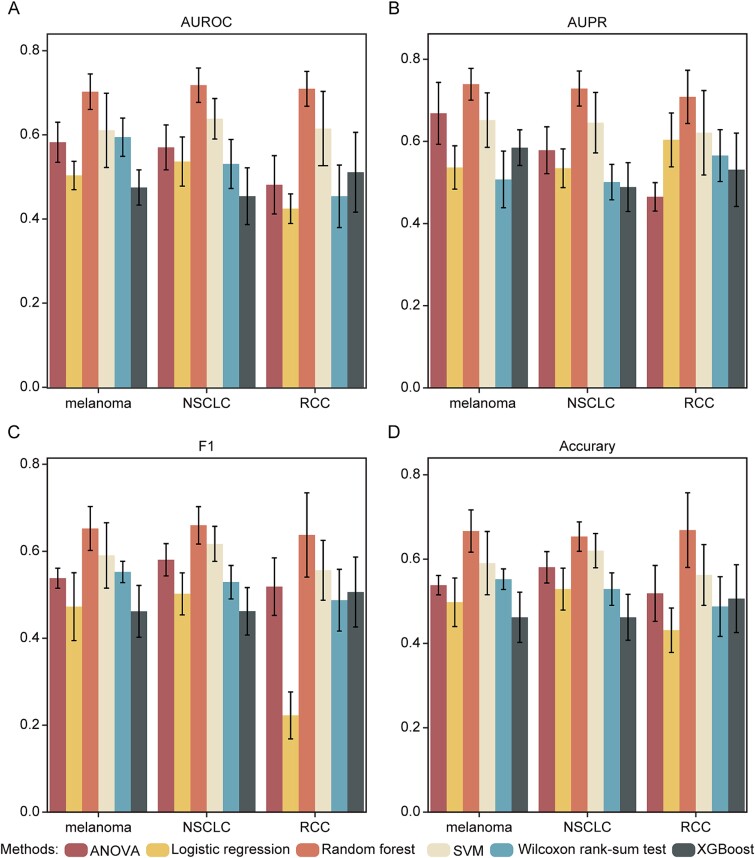
Performance comparison of DrugReSC with various feature importance calculation methods. A-D performance comparison of DrugReSC with various feature importance calculation methods including logistic regression, SVM, ANOVA, Wilcoxon rank-sum test, XGBoost, and random forest based on AUROC (A), AUPR (B), F1-score (C), and accuracy (D) metrics. The bar plots show the means of evaluation metrics with standard deviations. The error bars represent +/− one standard deviation, derived through n = 10 independent experiments.

The results depicted in Fig. 2 showed that DrugReSC combined with random forest achieved the highest average AUROC values across all three datasets: 0.702, 0.718, and 0.709, respectively. These values outperformed DrugReSC paired with SVM (0.611, 0.638, and 0.615), logistic regression (0.503, 0.536, and 0.424), ANOVA (0.582, 0.570, and 0.481), Wilcoxon test (0.594, 0.531, and 0.454), and XGBoost (0.475, 0.454, and 0.511) (Fig. 2A). The results obtained from metrics such as AUPR, F1-score, and accuracy are consistent with those obtained from the AUROC metric. (Fig. 2B-D and Supplementary Tables 5–7). In summary, it was clear that DrugReSC, when coupled with random forest, consistently demonstrated superior performance in predicting candidate drugs. Hence, we opted for the random forest method in the subsequent analysis, while retaining other methods as potential inputs for DrugReSC.

### DrugReSC outperforms both bulk and single-cell data-based methods

**Figure 3:**
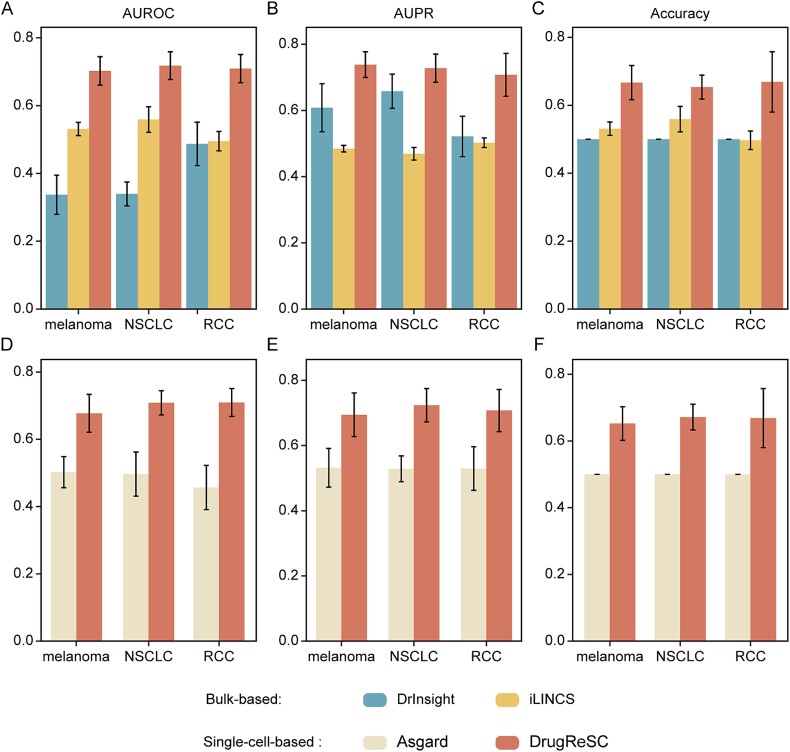
Comparison of DrugReSC with bulk- and single-cell data-based drug repurposing methods. A-C comparison of DrugReSC with bulk-sample-based drug repurposing methods DrInsight and iLINCS based on AUROC (A), AUPR (B), and accuracy (C) metrics. D-F comparison of DrugReSC with a single-cell data-based drug repurposing method Asgard based on AUROC (D), AUPR (E), and accuracy (F) metrics.

Most existing methods for drug repurposing are based on bulk RNA-seq data. This study aims to demonstrate the enhanced performance and insights gained by leveraging single-cell resolution data for drug repurposing. To highlight these advantages, we implemented DrInsight [30] and iLINCS [22] on three TCGA datasets (TCGA-SKCM, TCGA-LUAD, TCGA-KIRC), comparing their predictive capabilities with those of DrugReSC using the same ground lists (Methods). Compared with DrInsight (average AUROC: 0.337, 0.339, and 0.487) and iLINCS (average AUROC: 0.531, 0.559, and 0.495) methods, DrugReSC maintained an advantage of at least 20% (Fig. 3A). The consistent findings were further validated by the AUPR and accuracy results (Fig. 3B, C and Supplementary Tables 8-10). Overall, DrugReSC outperformed other drug repurposing methods based on bulk samples.

We also conducted a comparative analysis between DrugReSC and ASGARD, a drug repurposing method based on scRNA-seq data. Note that the ASGARD methodology required the inclusion of single-cell data from both tumor tissues and corresponding normal control tissues. However, DrugReSC had the advantage of allowing the exclusive utilization of single-cell data from diseased tissues, with the flexibility of whether or not to include single-cell data from normal tissues. For a fair comparison, we merged the corresponding single-cell datasets of NSCLC and melanoma with the single-cell data from their respective normal control tissues to assess the performance of the two methods [41, 42]. Due to the inclusion of single-cell data from normal tissues within the RCC single-cell dataset [40], no additional processing was required. The outcomes demonstrated the superior performance of DrugReSC, with average AUROC values of 0.677, 0.708, and 0.709 for melanoma, NSCLC, and RCC, respectively. In comparison, the AUROC values obtained by the ASGARD method were 0.502, 0.497, and 0.457 for the corresponding cancer types (Fig. 3D). The consistent findings persisted across the results obtained using AUPR and accuracy as measurement metrics (Fig. 3E, F and Supplementary Tables 11-13). These results validated that DrugReSC exhibited a higher accuracy in drug prediction compared to the currently published ASGARD method, which was based on scRNA-seq data for drug repurposing.

**Figure 4:**
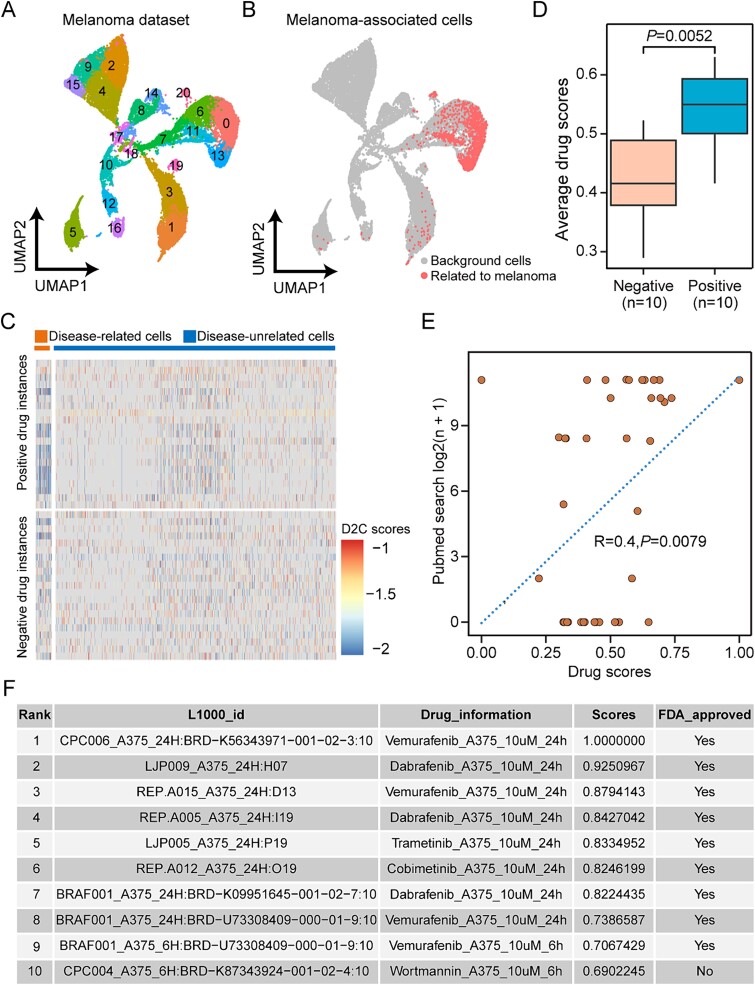
Drug repurposing in melanoma patient samples. (A) the UMAP plot of single-cell data in the melanoma case. (B) the UMAP visualization of PACSI-identified cells. (C) Heatmap displaying the D2C scores of positive and negative drug instances between melanoma-related cells and other cells. This heatmap is one of 10 visualizations representing the drug-by-cell matrix generated by DrugReSC, with the remaining nine heatmaps presented in Supplementary Fig. 4. (D) box plot showing the average drug scores of positive and negative drugs within the 10 DrugReSCpredicted candidate drug lists for melanoma. A two-sided Wilcoxon rank-sum test was performed to estimate the significance level. (E) Pearson correlation between drug importance scores predicted by DrugReSC for melanoma and the corresponding number of search results (log2(n + 1)) on PubMed. This correlation plot is one of 10 generated to illustrate the association between DrugReSC results and available supporting evidence from the literature, with the remaining nine plots provided in Supplementary Fig. 5. (F) table of the top 10 candidate drugs identified by DrugReSC for melanoma, including the ID of the L1000 project for each drug instance, treatment information, drug score, and FDA approval status. This table is one of 10 tables representing the output candidate drug lists generated by DrugReSC for melanoma, with complete results reported in Supplementary Table 14.

**Figure 5:**
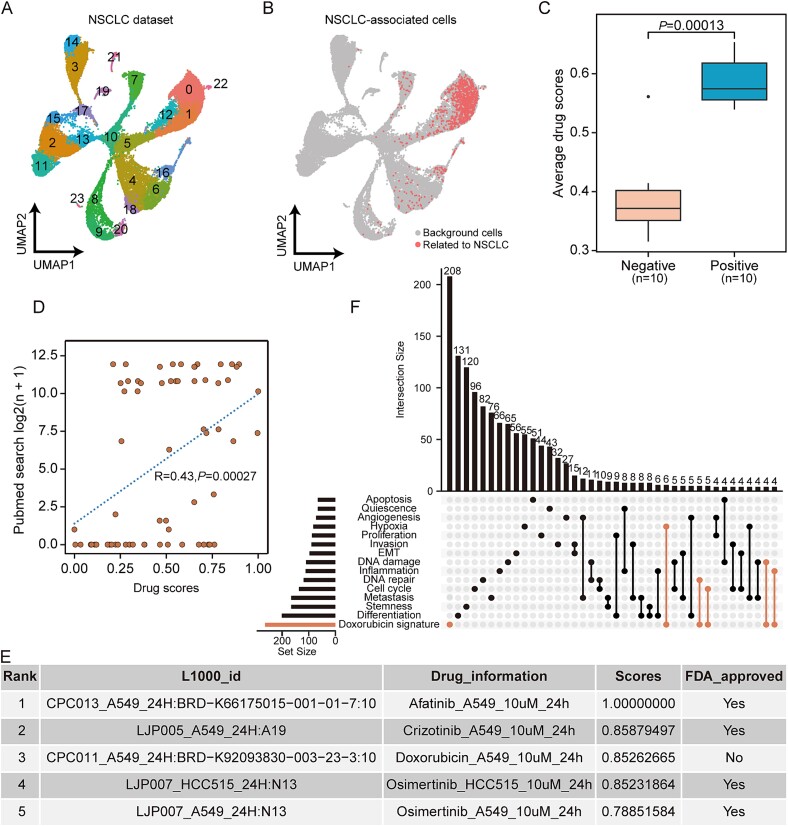
Drug repurposing for non-small cell carcinoma case. (A) the UMAP plot of single-cell data from NSCLC patients. (B) the UMAP visualization of PACSI-identified cells. (C) box plot showing the average drug scores of positive and negative drugs within the 10 DrugReSC-predicted candidate drug lists for NSCLC. A two-sided Wilcoxon rank-sum test was performed to estimate the significance level. (D) Pearson correlation between drug importance scores predicted by DrugReSC for NSCLC and the corresponding number of search results (log2(n + 1)) on PubMed. This correlation plot is one of 10 generated to illustrate the association between DrugReSC results and available supporting evidence from the literature, with the remaining nine plots provided in Supplementary Fig. 6. (E) table of the top 10 candidate drugs identified by DrugReSC for NSCLC, including the ID of the L1000 project for each drug instance, treatment information, drug score, and FDA approval status. This table is one of 10 tables representing the output candidate drug lists generated by DrugReSC for NSCLC, with complete results reported in Supplementary Table 15. (F) UpSet plot detailing overlaps between the doxorubicin signature and known cancer-associated gene sets from the CancerSEA database.

### Drug repurposing for melanoma

Melanoma, recognized as the most aggressive and lethal type of skin cancer, posed a significant challenge in cancer treatment [43]. In this study, we employed DrugReSC to explore potential therapeutic options for this malignancy. We initially applied the PACSI method, guided by bulk samples in TCGA-SKCM, to identify melanoma-associated cells within the scRNA-seq dataset obtained from patients with acral melanoma [31]. Among 24,659 cells from different cell types (Fig. 4A), 1315 cells were selected by PACSI, which were linked to melanoma pathogenesis (Fig. 4B). After that, we displayed a heatmap of the drug-by-cell matrix constructed by DrugReSC, illustrating pronounced differences in D2C scores among cells with two phenotypes in positive drugs as compared to negative drugs (Fig. 4C and Supplementary Fig. 4). We also conducted the Wilcoxon rank-sum test to compare the importance scores of positive and negative drugs in the DrugReSC results. Our analysis revealed significantly higher importance of positive drugs in melanoma compared to negative drugs (Fig. 4D). To investigate the correlation between these candidate drugs and melanoma, we employed the RISmed method [37], seeking supporting evidence from studies reported in the PubMed database. We observed a significant positive correlation between the drug scores predicted by DrugReSC and the number of results obtained from the PubMed search (Fig. 4E and Supplementary Fig. 5). Drugs with higher DrugReSC-predicted importance scores, indicating a higher rank, were associated with a greater number of published studies related to melanoma. For example, as depicted in Fig. 4F, one of the 10 melanoma candidate drug lists predicted by DrugReSC was shown (Supplementary Table 14). Among the top 10 drugs in the list, nine candidate drugs had received FDA approval for melanoma, while the tenth candidate drug wortmannin had not been approved by the FDA. Wortmannin, a metabolite produced by fungi, acted as a targeted inhibitor of the phosphatidylinositol 3-kinase family [44]. Numerous studies have demonstrated the antitumor effects of wortmannin in human and mouse melanoma cell models [45–47].

**Figure 6:**
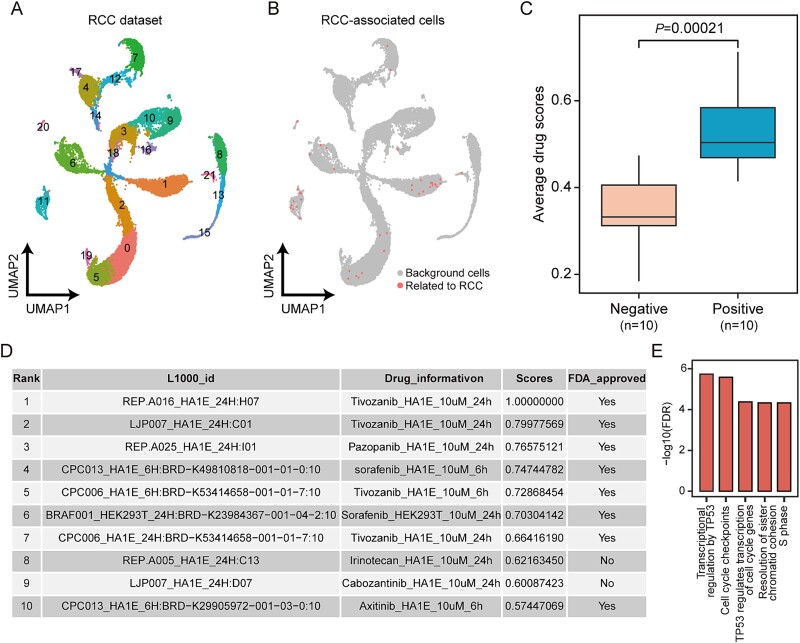
Drug repurposing for renal cell carcinoma patients. (A) the UMAP plot of single-cell data in the RCC case. (B) the UMAP visualization of PACSI-identified cells. (C) box plot showing the average drug scores of positive and negative drugs within the 10 DrugReSC-predicted candidate drug lists for RCC. A two-sided Wilcoxon rank-sum test was performed to estimate the significance level. (D) table of the top 10 candidate drugs identified by DrugReSC for RCC, including the ID of the L1000 project for each drug instance, treatment information, drug score, and FDA approval status. This table is one of 10 tables representing the output candidate drug lists generated by DrugReSC for RCC, with complete results reported in Supplementary Table 16. (E) the top five Reactome pathways enriched for genes in irinotecan signature.

### Drug repurposing for non-small cell lung cancer

To identify candidate drugs that could reduce mortality in NSCLC, we obtained scRNA-seq data from five fresh and frozen NSCLC tumor samples [39]. The dataset comprised 36,584 cells, with 1051 cells identified by DrugReSC, leveraging information from bulk data from TCGA-LUAD, as closely associated with NSCLC (Fig. 5A, B). To validate the predictive outcomes of the DrugReSC method, we computed the DrugReSC scores for positive and negative drugs. The results revealed a significant elevation in the DrugReSC scores for positive drugs compared to negative drugs in the context of NSCLC (Fig. 5C). Furthermore, we also noted a positive correlation where drugs displaying higher DrugReSC-predicted scores, indicative of a superior rank, were linked to an increased number of published studies related to NSCLC (Fig. 5D and Supplementary Fig. 6). In the ranked list of candidate drugs predicted by DrugReSC for NSCLC, doxorubicin (3rd) drew our attention, as it was the only drug among the top five that had not received FDA approval for NSCLC treatment (Fig. 5E and Supplementary Table 15). Doxorubicin originated from a secondary metabolite produced by *Streptomyces peucetius* [48]. In vitro cytotoxicity assays conducted on A549 human lung adenocarcinoma cells showed that co-administration of doxorubicin and paclitaxel synergistically induced cancer cell apoptosis and significantly reduced tumor size [49]. Therefore, we further explored the potential molecular mechanisms of doxorubicin by comparing its drug signatures with cancer-related functional gene sets from the CancerSEA database [33]. The results revealed that doxorubicin showed considerable intersections with hypoxia, DNA repair, and cell cycle functions, suggesting their potential impact on cancer-associated biological processes (Fig. 5F).

### Drug repurposing for renal cell carcinoma

RCC affects ~330,000 individuals globally each year, with an estimated annual mortality rate exceeding 140,000 among diagnosed RCC patients [50]. To identify candidate drugs for RCC, we initially obtained a single-cell dataset derived from the single-cell sequencing of two clear-cell RCC tumor samples and one normal kidney tissue sample [40] (Fig. 6A). By leveraging single-cell data and bulk mRNA expression profiles from TCGA-KIRC, we identified 44 cells associated with RCC (Fig. 6B). To validate the predictive results obtained through the DrugReSC method, we computed DrugReSC-predicted scores for both positive and negative drugs. The analysis revealed a substantial increase in the scores of positive drugs relative to negative drugs within RCC (Fig. 6C). Upon reviewing the ranked list of candidate drugs predicted by DrugReSC, our attention was drawn to irinotecan (8th), which stands out as one of only two drugs among the top 10 candidates not FDA-approved for treating RCC (Fig. 6D and Supplementary Table 16). Irinotecan, a camptothecin derivative, had demonstrated antitumor efficacy against diverse solid tumors, including colorectal, pancreatic, ovarian, and lung cancers [51, 52]. Recent researches have indicated a notable survival advantage of Irinotecan in human late-stage RCC models [53, 54]. To investigate the potential molecular mechanisms underlying the action of irinotecan on RCC, we conducted a functional enrichment analysis on its signature. We observed that irinotecan primarily modulated genes involved in TP53 transcription and cell cycle-related processes, both of which played a role in RCC pathogenesis [55] (Fig. 6E and Supplementary Table 17).

## Discussion

Considering the high heterogeneity of cancer, personalized medicine emerges as a future trend in oncology treatment. Its focus lies in tailoring appropriate therapeutic strategies to each patient based on the genomic characteristics of their tumor. In this study, we introduce DrugReSC, a novel drug repurposing method based on single-cell RNA sequencing data, aimed at targeting specific cell subpopulations crucial for disease pathology. By integrating single-cell transcriptomic data of diseases and drug signatures, DrugReSC constructs a drug-by-cell matrix delineating the relationship between each drug and each cell. Machine learning algorithms are then employed to compute the drug signatures crucial for cell phenotype classification. To assess the performance of DrugReSC in drug repurposing, we compare it with existing drug repurposing methods based on bulk transcriptome data and those based on single-cell transcriptome data across multiple cancer cases. The results indicate that DrugReSC outperforms these methods in drug prediction, with the candidate drugs it predicts for melanoma, NSCLC, and RCC garnering strong support in the literature.

We attempted various statistical and machine learning methods, including SVMs, random forests, analysis of variance, logistic regression, XGBoost, and the Wilcoxon rank-sum test, to assess the contribution of drugs to cell phenotype changes. Our findings indicated that the random forest model combined with perturbation-based methods was most suitable for evaluating the performance of DrugReSC. Moreover, DrugReSC was designed as an adaptable framework, capable of accommodating different methods to meet the diverse needs of users. Additionally, we explored a voting strategy where a drug is considered a candidate if at least three out of six methods vote for it. The results indicated that the voting strategy performed worse than the random forest method, possibly due to the lack of integration and synergy among the models (Supplementary Tables 18–20).

We also compared several methods for identifying disease-relevant cells, such as Scissor, DEGAS, and PACSI, to assess their effect on DrugReSC’s performance. The results revealed that the DrugReSC framework, integrated with the PACSI method, exhibited optimal and robust performance across several cases (Supplementary Fig. 7A–D and Supplementary Tables 21–23). Additionally, we explored various methods for constructing the drug-by-cell matrix, including ssGSEA, AUCell [56], and UniPath [57]. The results indicated that utilizing ssGSEA for constructing the drug-by-cell matrix led to the optimal performance of DrugReSC (Supplementary Fig. 7E–H and Supplementary Tables 24–26). Therefore, we set ssGSEA as the default method, allowing users to select from the other two options.

Given the high dropout rates in scRNA-seq data, we investigated the impact of missing value imputation on the performance of DrugReSC. We applied the MAGIC method to impute the single-cell gene expression matrices for melanoma, NSCLC, and RCC [58]. We observed a decrease in the performance of the DrugReSC method for predicting candidate drugs after imputing single-cell RNA-seq data (Supplementary Tables 27–29). We hypothesize that this decrease may be due to the imputation process introducing additional noise and erroneous signals. Furthermore, the imputation method might smooth out biologically important heterogeneity between cells, rendering DrugReSC less effective in capturing drug effect-related features. This finding underscores the necessity of carefully selecting and evaluating imputation methods when applying DrugReSC to ensure the accuracy and reliability of downstream analyses.

To further examine the in vitro antitumor activity of the cancer candidate drugs identified by DrugReSC (wortmannin for melanoma, doxorubicin for NSCLC, irinotecan for RCC), we analyzed the drug response data from the Genomics of Drug Sensitivity in Cancer (GDSC) database [59]. Due to the absence of wortmannin response data in the skin cutaneous melanoma cell lines within the GDSC database, we compared the IC50 distributions of doxorubicin and irinotecan in lung adenocarcinoma and renal clear cell carcinoma cell lines, respectively, with those of 10 randomly selected drugs. Of note, both doxorubicin and irinotecan exhibited strong antitumor activity, with significantly lower IC50 values compared to the randomly selected drugs (Supplementary Fig. 8).

To explore the composition of cancer-associated cells identified by DrugReSC, we analyzed the cell type proportions in the identified cells for three cases. The results indicated that malignant tumor cells constituted the largest proportion, exceeding 50% (Supplementary Fig. 9). Additionally, the identified cells included a minor portion of other significant cells, such as immune cells, fibroblasts, and endothelial cells, which are key components of the tumor microenvironment. These microenvironmental cells also play crucial roles in cancer drug therapy; however, their specific functions and mechanisms require further investigation. Furthermore, an intriguing observation is that 99% of the cells associated with the melanoma phenotype are melanocytes, which reflects the unique biological characteristics of melanoma. Melanoma originates from melanocytes, the pigment-producing cells in the skin [60]. This strong lineage relationship likely explains why melanocytes dominate the phenotype-associated cells in our analysis. One potential reason for the low presence of immune cells and other microenvironmental components among the melanoma phenotype-associated cells could be the inherent genetic mutations, such as the BRAF mutation, in melanoma cells that drive their low immune cell infiltration and independent growth [61–63].

Although the DrugReSC method has shown high prediction performance, it still has several potential limitations. We solely computed the importance scores of each drug for cellular phenotype changes without exploring the directionality of drug effects on cellular phenotypic changes. We intend to extend our methodology to investigate whether the impact of drugs on cellular phenotypes is a positive promotion or reverse inhibition in future studies. Additionally, as a computational method, the predictive outcomes of DrugReSC require validation through in vitro and in vivo experimental animal models, as well as clinical trials, before the clinical application can be considered.

In summary, this study demonstrates the exceptional performance of DrugReSC in drug repurposing by targeting disease-relevant cells. We envision our work as a driving force for advancing the utilization of single-cell sequencing data in developing drug repurposing algorithms for personalized medicine. Furthermore, our research provides insights that could assist pharmaceutical researchers in identifying viable therapeutic strategies for currently untreatable diseases.

**Key Points**

- DrugReSC identifies candidate drugs by specifically targeting key cellular subpopulations of diseases, addressing a crucial gap in the field of drug repurposing.
- DrugReSC leverages single-cell RNA-seq data to overcome tissue heterogeneity, promoting the development of drug repurposing algorithms based on single-cell data.
- The DrugReSC method has been developed into an R package, enabling researchers to conveniently identify viable treatment strategies for currently incurable diseases.

## Supplementary Material

Supplementary_bbae490

## Data Availability

Single-cell RNA sequencing data are accessible from GEO (Accession number: ‘GSE189889’, ‘GSE140819’, ‘GSE152938’, ‘GSE183047’, and ‘GSE122960’). Bulk gene expression data, along with clinical data, are available from the UCSC Xena database (http://xena.ucsc.edu/). Information on drugs approved by the FDA for each disease was obtained from the DrugBank database (https://go.drugbank.com/). Drug-induced gene expression profiles generated by the LINCS L1000 project were extracted from the iLINCS database (http://www.ilincs.org/ilincs/).

## Code availability

The code for the DrugReSC method is accessible as an R package repository on the GitHub platform (https://github.com/Chonghui-Liu/DrugReSC).
