# Supplementary material for: DrugReSC: targeting disease-critical cell subpopulations with single-cell transcriptomic data for drug repurposing in cancer
Source: Brief Bioinform. 2024 Sep 30;25(6):bbae490. doi: 10.1093/bib/bbae490 (PMC11442150; doi:10.1093/bib/bbae490)
Supplement: Supplementary_bbae490 [file supplementary_bbae490.zip › Supplementary Information_Revised_Draft_2.docx]

**Supplementary Materials:**

DrugReSC: Targeting disease-critical cell subpopulations with single-cell transcriptomic data for drug repurposing in cancer

**Authors:**

Chonghui Liu^1, 2^, Yan Zhang^3, 4^, Yingjian Liang^5^, Tianjiao Zhang^2^*, and Guohua Wang^2^*

^1^ College of Life Science, Northeast Forestry University, Harbin 150040, China.

^2^ College of Computer and Control Engineering, Northeast Forestry University, Harbin 150040, China.

^3^ Kunming Institute of Zoology, Chinese Academy of Sciences, Kunming 650201, Yunnan, China.

^4^ University of Chinese Academy of Sciences, Beijing 100049, China.

^5^ Department of General Surgery, the First Affiliated Hospital of Harbin Medical University, Harbin 150007, China.

***Corresponding authors:**

Tianjiao Zhang. E-mail: tianjiaozhang@nefu.edu.cn; Guohua Wang. E-mail: ghwang@nefu.edu.cn.

**This PDF file includes:**

**Supplementary Figure 1.** Performance of random forest model with varying numbers of trees in melanoma cases.

**Supplementary Figure 2.** Performance of random forest model with varying numbers of trees in NSCLC cases.

**Supplementary Figure 3.** Performance of random forest model with varying numbers of trees in RCC cases.

**Supplementary Figure 4.** The remaining nine heatmaps of D2C scores computed using DrugReSC of positive and negative drug instances between melanoma-related cells and the other cells.

**Supplementary Figure 5.** The remaining nine plots depict Pearson correlation between DrugReSC-predicted drug importance scores for melanoma and PubMed search results (log2(n + 1)).

**Supplementary Figure 6.** The remaining nine plots depict Pearson correlation between DrugReSC-predicted drug importance scores for NSCLC and PubMed search results (log2(n + 1)).

**Supplementary Figure 7.** Performance comparison of DrugReSC with alternative methods.

**Supplementary Figure 8.** Comparison of activity distribution between DrugReSC-identified candidate drugs and randomly selected drugs.

**Supplementary Figure 9.** The distribution of PACSI-identified cells by cell types.

**Supplementary Table 5.** Comparison of feature importance calculation methods on melanoma dataset

**Supplementary Table 6.** Comparison of feature importance calculation methods on NSCLC dataset.

**Supplementary Table 7.** Comparison of feature importance calculation methods on RCC dataset.

**Supplementary Table 8.** Comparison of DrugReSC with bulk data-based methods on melanoma dataset.

**Supplementary Table 9.** Comparison of DrugReSC with bulk data-based methods on NSCLC dataset.

**Supplementary Table 10.** Comparison of DrugReSC with bulk data-based methods on RCC dataset.

**Supplementary Table 11.** Comparison of DrugReSC with single-cell data-based methods on the integrated melanoma and normal skin tissue dataset.

**Supplementary Table 12.** Comparison of DrugReSC with single-cell data-based methods on the integrated NSCLC and normal lung tissue dataset.

**Supplementary Table 13.** Comparison of DrugReSC with single-cell data-based methods on RCC dataset.

**Supplementary Table 18.** Comparison of DrugReSC and voting strategy on melanoma dataset

**Supplementary Table 19.** Comparison of DrugReSC and voting strategy on NSCLC dataset

**Supplementary Table 20.** Comparison of DrugReSC and voting strategy on RCC dataset

**Supplementary Table 21.** Comparison of PACSI with alternative methods on melanoma dataset.

**Supplementary Table 22.** Comparison of PACSI with alternative methods on NSCLC dataset.

**Supplementary Table 23.** Comparison of PACSI with alternative methods on RCC dataset.

**Supplementary Table 24.** Comparison of ssGSEA with alternative methods on melanoma dataset.

**Supplementary Table 25.** Comparison of ssGSEA with alternative methods on NSCLC dataset.

**Supplementary Table 26.** Comparison of ssGSEA with alternative methods on RCC dataset.

**Supplementary Table 27.** Comparison of DrugReSC method with and without imputation on melanoma dataset.

**Supplementary Table 28.** Comparison of DrugReSC method with and without imputation on NSCLC dataset.

**Supplementary Table 29.** Comparison of DrugReSC method with and without imputation on RCC dataset.


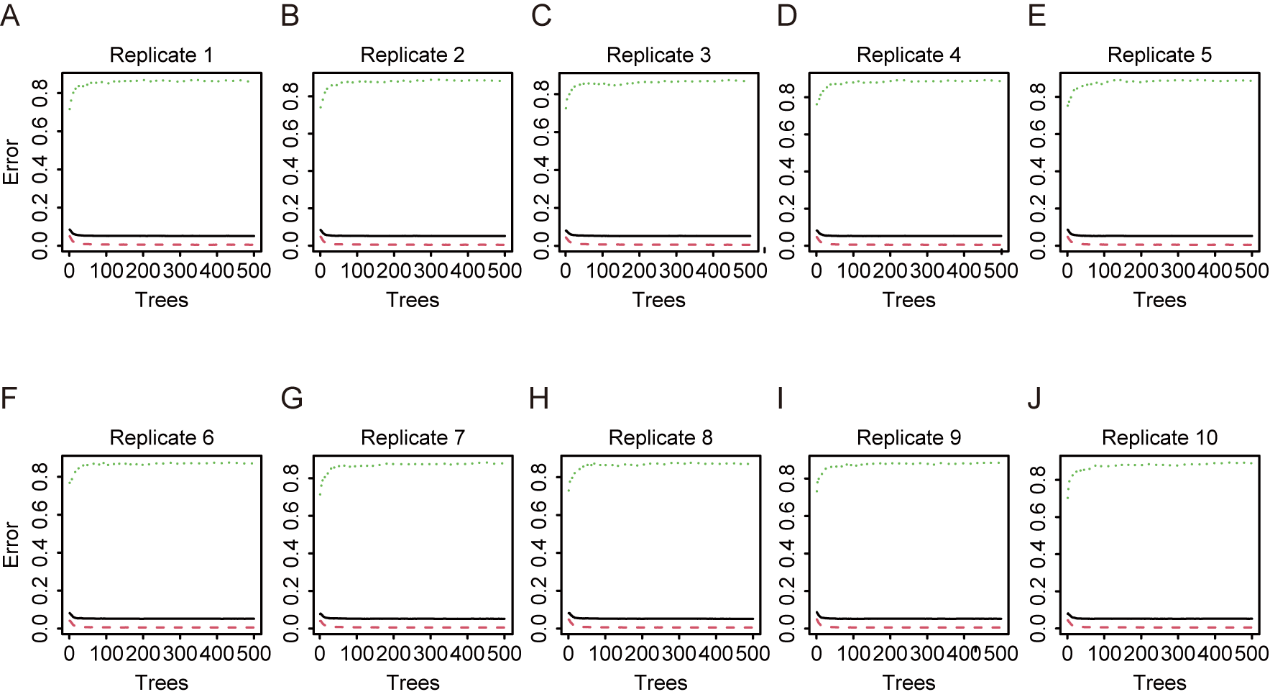


**Supplementary Figure 1.** Performance of random forest model with varying numbers of trees in melanoma cases. The x-axis represents the number of trees in the forest, while the y-axis represents the error rate.


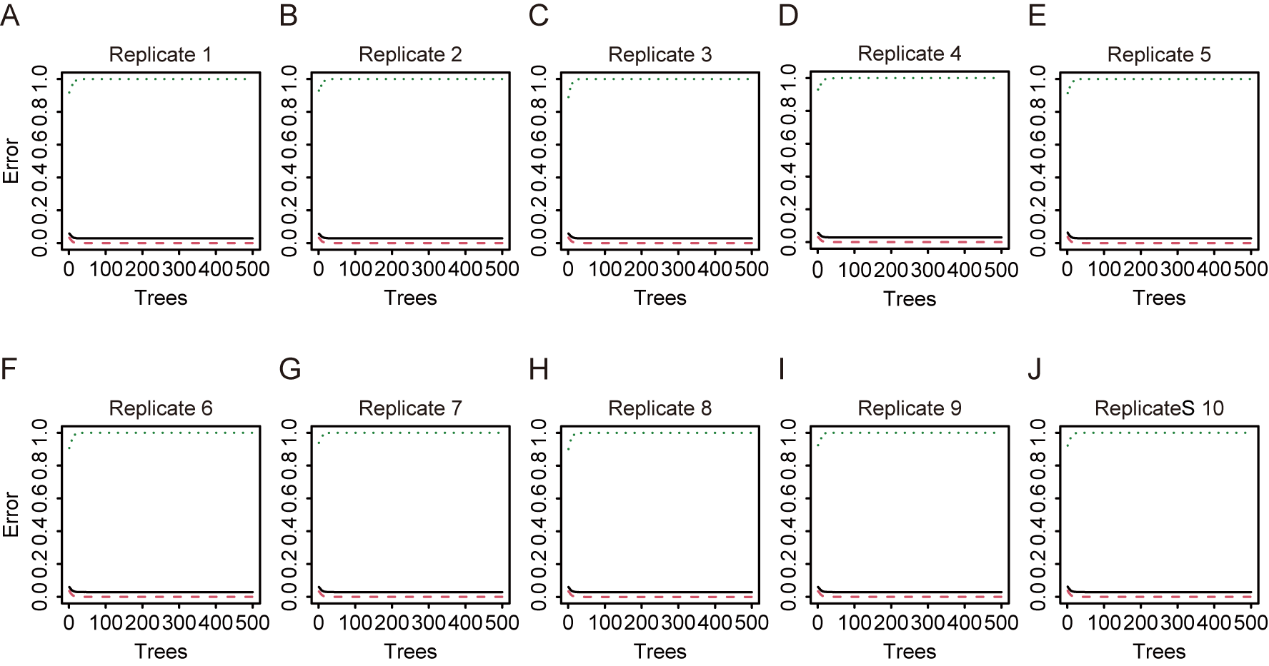


**Supplementary Figure 2.** Performance of random forest model with varying numbers of trees in NSCLC cases. The x-axis represents the number of trees in the forest, while the y-axis represents the error rate.

**
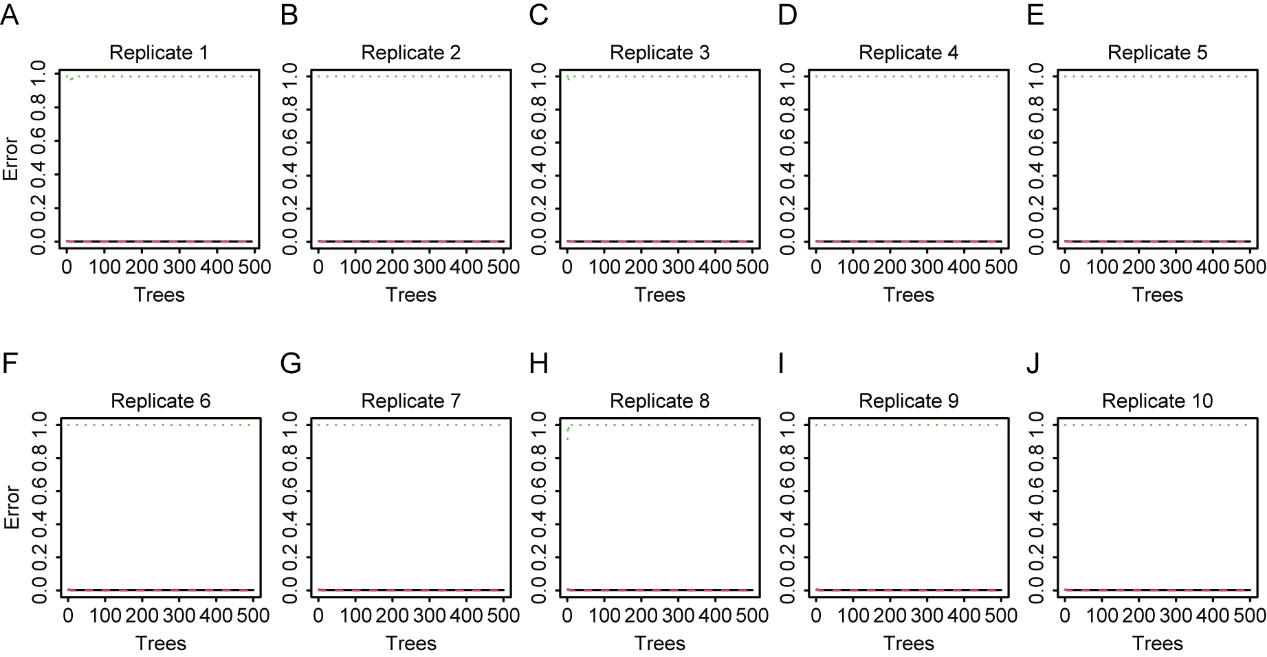
**

**Supplementary Figure 3.** Performance of random forest model with varying numbers of trees in RCC cases. The x-axis represents the number of trees in the forest, while the y-axis represents the error rate.

**
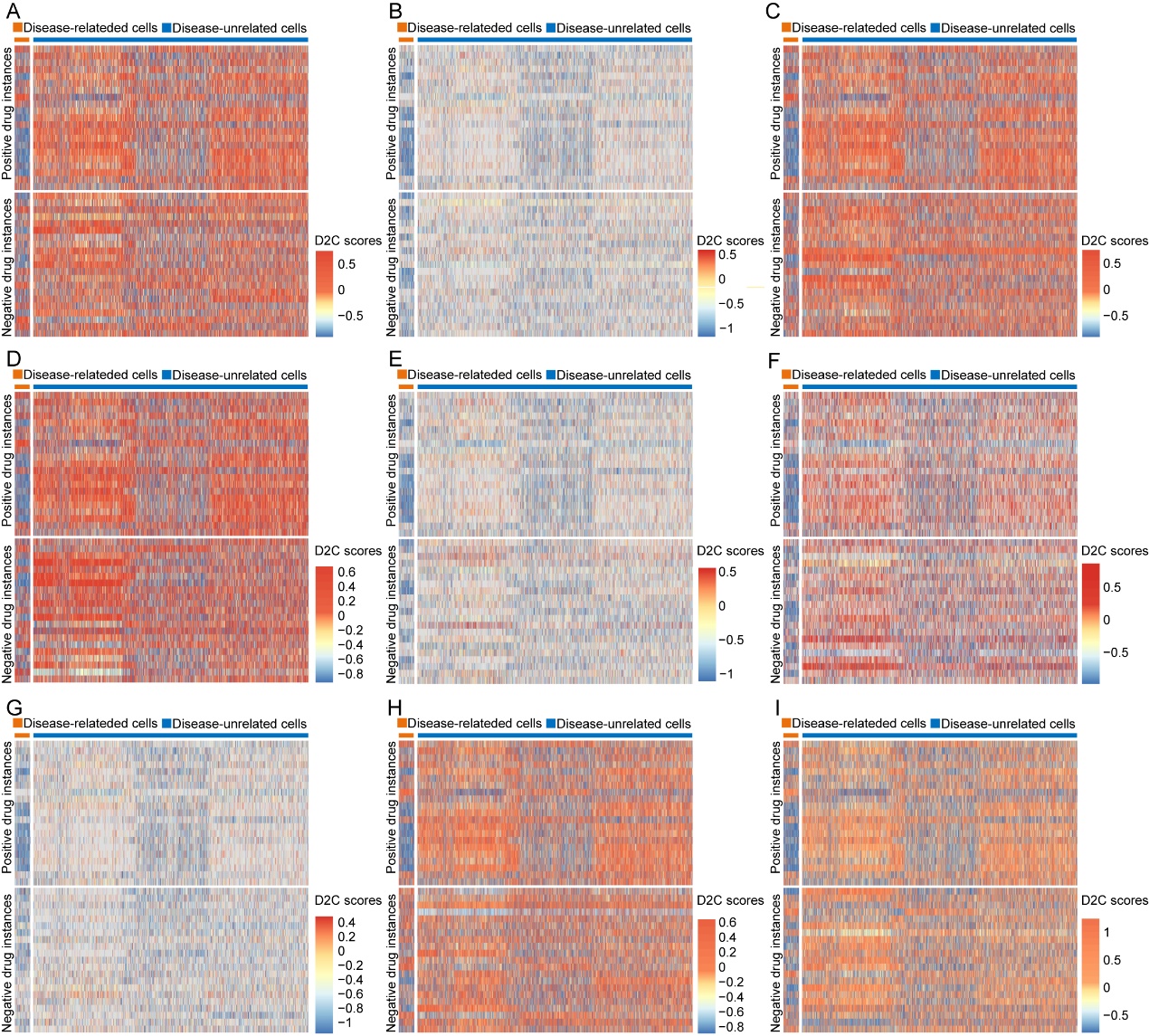
**

**Supplementary Figure 4.** The remaining nine heatmaps of D2C scores computed using DrugReSC of positive and negative drug instances between melanoma-related cells and the other cells. Results are derived from the remaining nine drug-by-cell matrices of melanoma case.


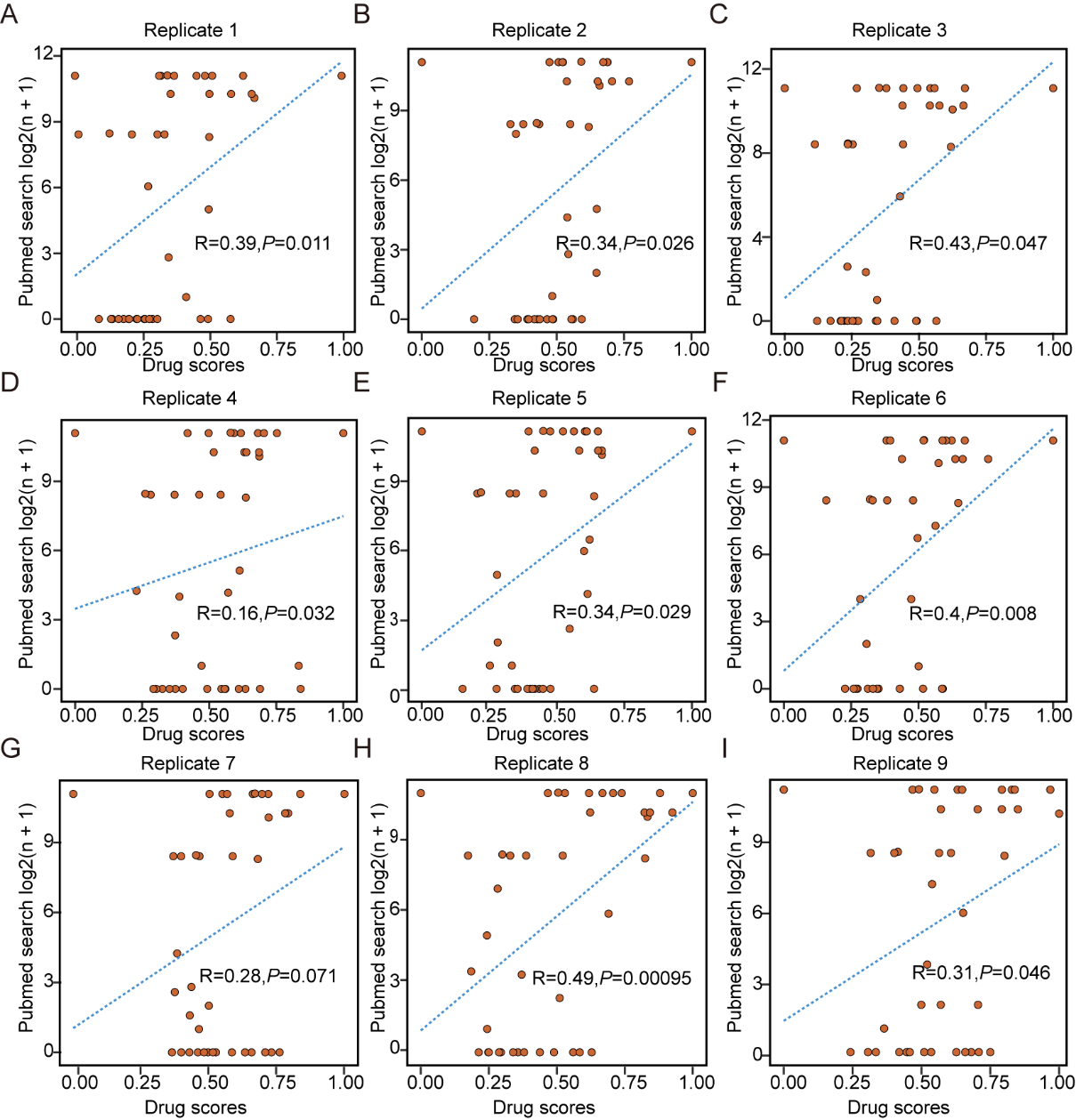


**Supplementary Figure 5.** The remaining nine plots depict Pearson correlation between DrugReSC-predicted drug importance scores for melanoma and PubMed search results (log2(n + 1)). Results are derived from the remaining nine candidate drug lists identified by DrugReSC for the melanoma case.


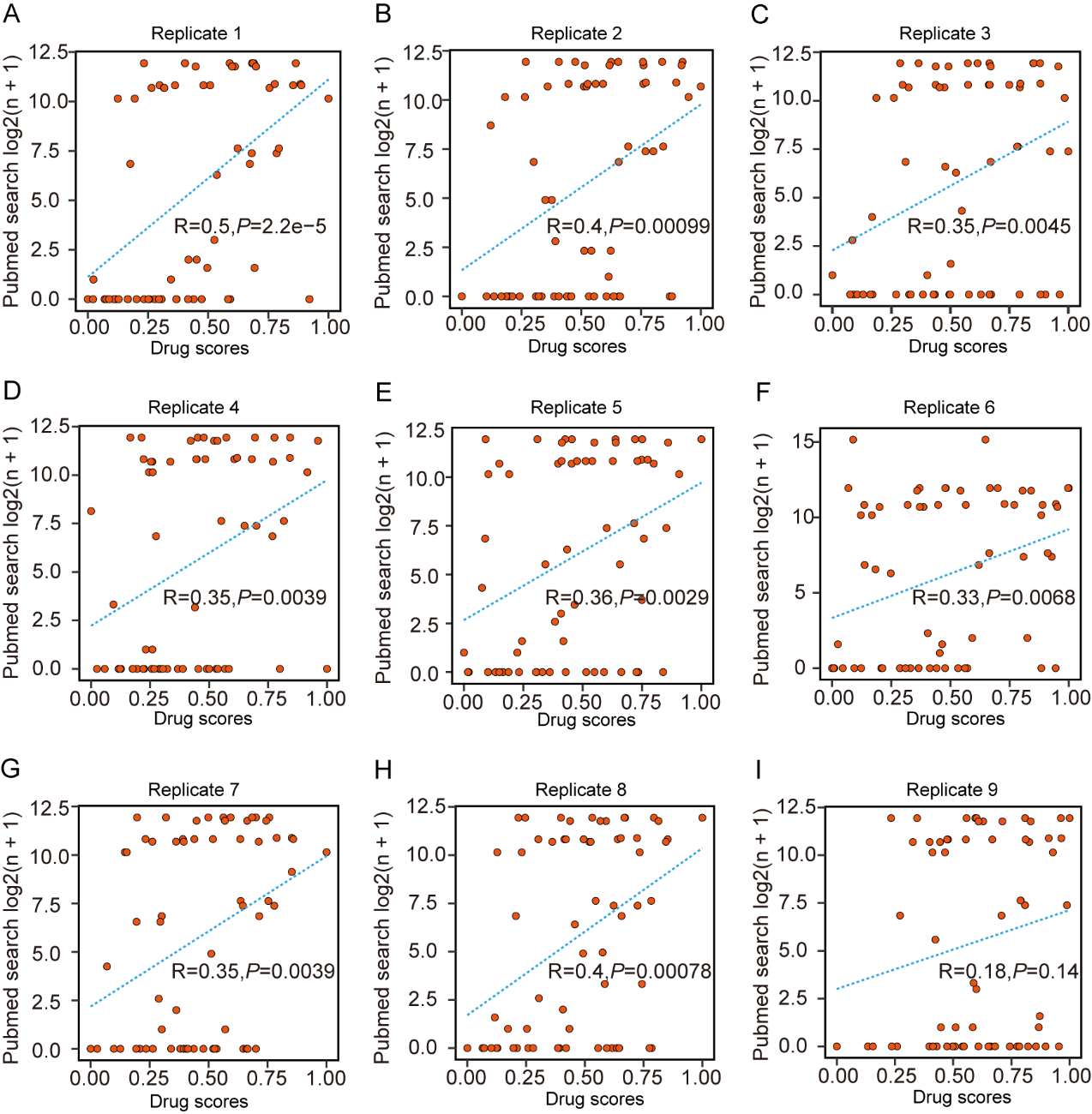


**Supplementary Figure 6.** The remaining nine plots depict Pearson correlation between DrugReSC-predicted drug importance scores for NSCLC and PubMed search results (log2(n + 1)). Results are derived from the remaining nine candidate drug lists identified by DrugReSC for the NSCLC case.

**
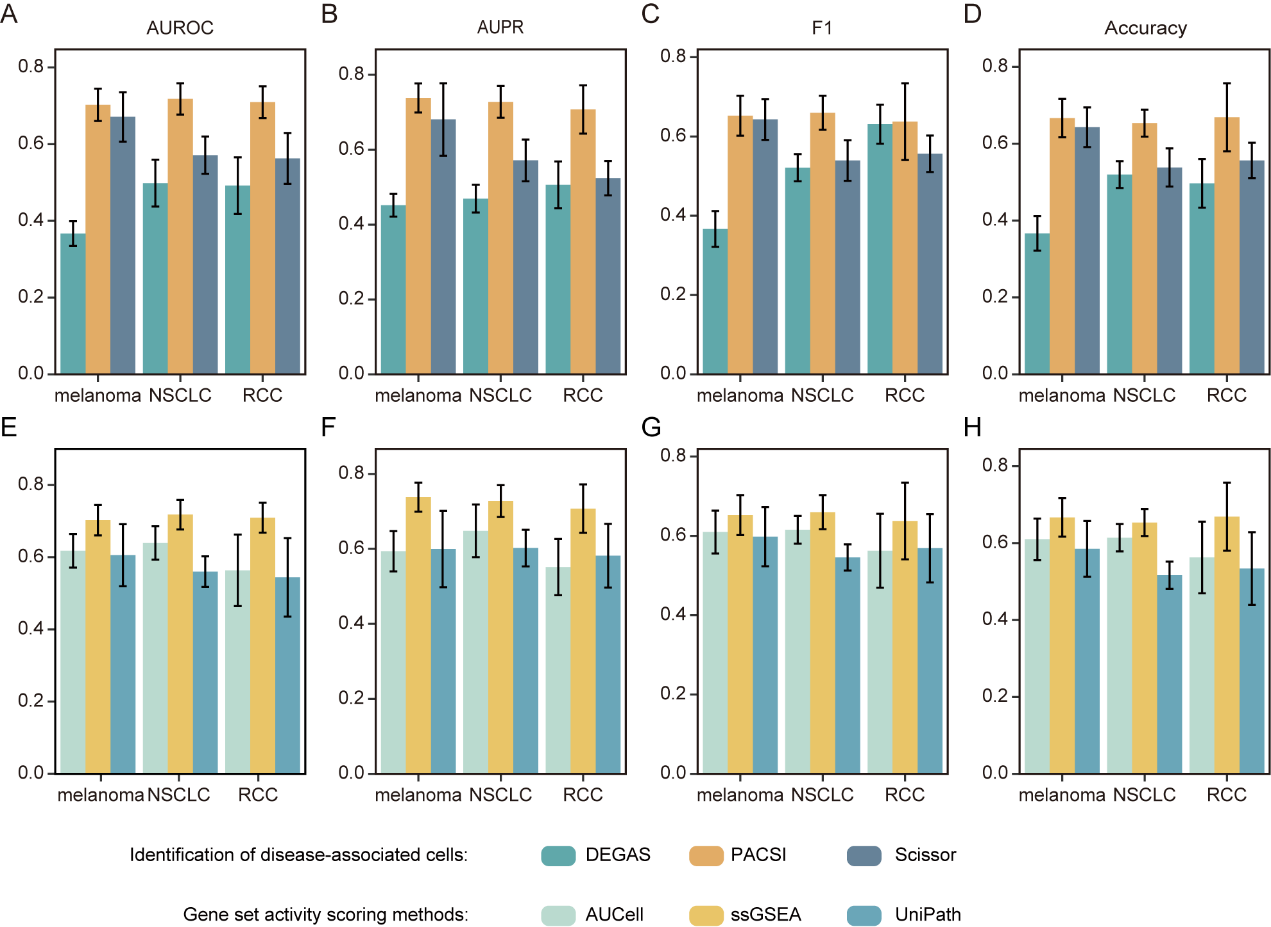
**

**Supplementary Figure 7. Performance comparison of DrugReSC with alternative methods. A-D** Comparison of DrugReSC with various disease-related cell identification methods including Scissor, DEGAS, and PACSI based on AUROC (**A**), AUPR (**B**), F1 (**C**), and accuracy (**D**) metrics. **E-H** Comparison of DrugReSC with various gene set activity scoring methods including AUCell, UniPath, and ssGSEA based on AUROC (**E**), AUPR (**F**), F1 (**G**), and accuracy (**H**) metrics.

**
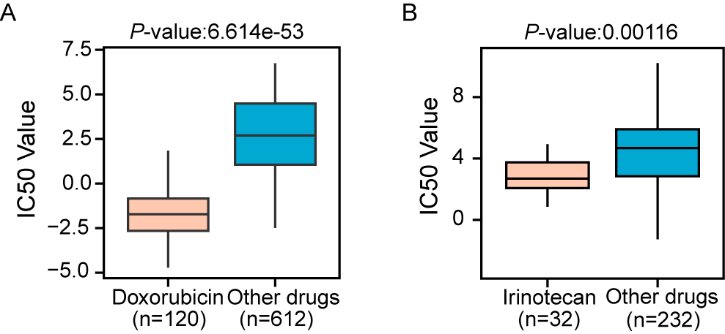
**

**Supplementary Figure 8.** Comparison of activity distribution between DrugReSC-identified candidate drugs and randomly selected drugs. **A** Comparison of compound activity distribution in NSCLC cell lines between candidate drug doxorubicin and 10 randomly selected drugs. **B** Comparison of compound activity distribution in RCC cell lines between candidate irinotecan and 10 randomly selected drugs. A two-sided Wilcoxon rank-sum test was performed to estimate the significance level.

**
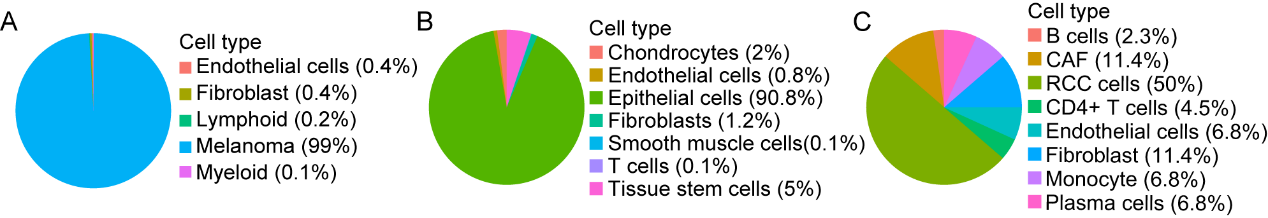
**

**Supplementary Figure 9.** The distribution of PACSI-identified cells by cell types. **A** The distribution of PACSI-identified cells associated with melanoma by cell type. **B** The distribution of PACSI-identified cells associated with NSCLC by cell type. **C** The distribution of PACSI-identified cells associated with RCC by cell type.

**Supplementary Table 5.** Comparison of feature importance calculation methods on melanoma dataset

| **Method** | **AUROC** | **AUPR** | **F1** | **Accuracy** |
| --- | --- | --- | --- | --- |
| Logistic Regression | 0.503±0.034 | 0.536±0.053 | 0.473±0.078 | 0.498±0.058 |
| SVM | 0.611±0.088 | 0.651±0.066 | 0.59±0.075 | 0.59±0.075 |
| ANOVA | 0.582±0.048 | 0.667±0.075 | 0.538±0.023 | 0.538±0.023 |
| Random Forest | 0.702±0.042 | 0.738±0.039 | 0.652±0.05 | 0.667±0.05 |
| Wilcoxon | 0.594±0.046 | 0.507±0.069 | 0.552±0.025 | 0.552±0.025 |
| XGBoost | 0.475±0.042 | 0.584±0.043 | 0.462±0.06 | 0.462±0.06 |

**Supplementary Table 6.** Comparison of feature importance calculation methods on NSCLC dataset

| **Method** | **AUROC** | **AUPR** | **F1** | **Accuracy** |
| --- | --- | --- | --- | --- |
| Logistic Regression | 0.536±0.058 | 0.534±0.047 | 0.502±0.048 | 0.529±0.05 |
| SVM | 0.638±0.048 | 0.645±0.074 | 0.617±0.04 | 0.62±0.041 |
| ANOVA | 0.57±0.053 | 0.578±0.057 | 0.581±0.037 | 0.581±0.037 |
| Random Forest | 0.718±0.041 | 0.728±0.042 | 0.66±0.043 | 0.653±0.035 |
| Wilcoxon | 0.531±0.058 | 0.5±0.043 | 0.529±0.039 | 0.529±0.039 |
| XGBoost | 0.454±0.067 | 0.488±0.059 | 0.462±0.055 | 0.462±0.055 |

**Supplementary Table 7.** Comparison of feature importance calculation methods on RCC dataset

| **Method** | **AUROC** | **AUPR** | **F1** | **Accuracy** |
| --- | --- | --- | --- | --- |
| Logistic Regression | 0.424±0.035 | 0.603±0.066 | 0.222±0.054 | 0.431±0.053 |
| SVM | 0.615±0.088 | 0.62±0.103 | 0.556±0.069 | 0.562±0.072 |
| ANOVA | 0.481±0.069 | 0.464±0.035 | 0.519±0.066 | 0.519±0.066 |
| Random Forest | 0.709±0.042 | 0.707±0.065 | 0.637±0.097 | 0.669±0.089 |
| Wilcoxon | 0.454±0.074 | 0.565±0.063 | 0.488±0.071 | 0.488±0.071 |
| XGBoost | 0.511±0.095 | 0.53±0.089 | 0.506±0.08 | 0.506±0.08 |

**Supplementary Table 8.** Comparison of DrugReSC with bulk data-based methods on melanoma dataset

| **Method** | **AUROC** | **AUPR** | **Accuracy** |
| --- | --- | --- | --- |
| iLINCS | 0.531±0.02 | 0.484±0.01 | 0.531±0.02 |
| DrInsight | 0.337±0.058 | 0.608±0.073 | 0.5±0 |
| DrugReSC | 0.702±0.042 | 0.738±0.039 | 0.667±0.05 |

**Supplementary Table 9.** Comparison of DrugReSC with bulk data-based methods on NSCLC dataset

| **Method** | **AUROC** | **AUPR** | **Accuracy** |
| --- | --- | --- | --- |
| iLINCS | 0.559±0.038 | 0.469±0.019 | 0.559±0.037 |
| DrInsight | 0.339±0.035 | 0.658±0.052 | 0.5±0 |
| DrugReSC | 0.718±0.041 | 0.728±0.042 | 0.653±0.035 |

**Supplementary Table 10.** Comparison of DrugReSC with bulk data-based methods on RCC dataset

| **Method** | **AUROC** | **AUPR** | **Accuracy** |
| --- | --- | --- | --- |
| iLINCS | 0.495±0.029 | 0.502±0.015 | 0.497±0.027 |
| DrInsight | 0.487±0.064 | 0.521±0.061 | 0.5±0 |
| DrugReSC | 0.709±0.042 | 0.707±0.065 | 0.669±0.089 |

**Supplementary Table 11.** Comparison of DrugReSC with single-cell data-based methods on the integrated melanoma and normal skin tissue dataset

| **Method** | **AUROC** | **AUPR** | **Accuracy** |
| --- | --- | --- | --- |
| Asgard | 0.502±0.046 | 0.532±0.06 | 0.5±0 |
| DrugReSC | 0.677±0.056 | 0.694±0.067 | 0.652±0.05 |

**Supplementary Table 12.** Comparison of DrugReSC with single-cell data-based methods on the integrated NSCLC and normal lung tissue dataset

| **Method** | **AUROC** | **AUPR** | **Accuracy** |
| --- | --- | --- | --- |
| Asgard | 0.497±0.066 | 0.529±0.04 | 0.5±0 |
| DrugReSC | 0.708±0.036 | 0.724±0.051 | 0.672±0.039 |

**Supplementary Table 13.** Comparison of DrugReSC with single-cell data-based methods on RCC dataset

| **Method** | **AUROC** | **AUPR** | **Accuracy** |
| --- | --- | --- | --- |
| Asgard | 0.457±0.066 | 0.53±0.067 | 0.5±0 |
| DrugReSC | 0.709±0.042 | 0.707±0.065 | 0.669±0.089 |

**Supplementary Table 18.** Comparison of DrugReSC and voting strategy on melanoma dataset

| **Method** | **F1** | **Accuracy** |
| --- | --- | --- |
| Voting | 0.562±0.043 | 0.588±0.039 |
| DrugReSC | 0.652±0.05 | 0.667±0.05 |

**Supplementary Table 19.** Comparison of DrugReSC and voting strategy on NSCLC dataset

| **Method** | **F1** | **Accuracy** |
| --- | --- | --- |
| Voting | 0.625±0.038 | 0.568±0.05 |
| DrugReSC | 0.66±0.043 | 0.653±0.035 |

**Supplementary Table 20.** Comparison of DrugReSC and voting strategy on RCC dataset

| **Method** | **F1** | **Accuracy** |
| --- | --- | --- |
| Voting | 0.553±0.051 | 0.541±0.055 |
| DrugReSC | 0.637±0.097 | 0.669±0.089 |

**Supplementary Table 21.** Comparison of PACSI with alternative methods on melanoma dataset

| **Method** | **AUROC** | **AUPR** | **F1** | **Accuracy** |
| --- | --- | --- | --- | --- |
| Scissor | 0.671±0.064 | 0.681±0.097 | 0.643±0.051 | 0.643±0.051 |
| DEGAS | 0.367±0.032 | 0.451±0.03 | 0.367±0.045 | 0.367±0.045 |
| PACSI | 0.702±0.042 | 0.738±0.039 | 0.652±0.05 | 0.667±0.05 |

**Supplementary Table 22.** Comparison of PACSI with alternative methods on NSCLC dataset

| **Method** | **AUROC** | **AUPR** | **F1** | **Accuracy** |
| --- | --- | --- | --- | --- |
| Scissor | 0.571±0.049 | 0.571±0.056 | 0.539±0.051 | 0.538±0.05 |
| DEGAS | 0.498±0.061 | 0.469±0.037 | 0.521±0.034 | 0.52±0.035 |
| PACSI | 0.718±0.041 | 0.728±0.042 | 0.66±0.043 | 0.653±0.035 |

**Supplementary Table 23.** Comparison of PACSI with alternative methods on RCC dataset

| **Method** | **AUROC** | **AUPR** | **F1** | **Accuracy** |
| --- | --- | --- | --- | --- |
| Scissor | 0.563±0.066 | 0.524±0.046 | 0.556±0.046 | 0.556±0.046 |
| DEGAS | 0.492±0.074 | 0.506±0.063 | 0.631±0.049 | 0.497±0.063 |
| PACSI | 0.709±0.042 | 0.707±0.065 | 0.637±0.097 | 0.669±0.089 |

**Supplementary Table 24.** Comparison of ssGSEA with alternative methods on melanoma dataset

| **Method** | **AUROC** | **AUPR** | **F1** | **Accuracy** |
| --- | --- | --- | --- | --- |
| AUCell | 0.618±0.046 | 0.594±0.054 | 0.61±0.054 | 0.61±0.054 |
| UniPath | 0.605±0.086 | 0.6±0.102 | 0.598±0.074 | 0.585±0.072 |
| ssGSEA | 0.702±0.042 | 0.738±0.039 | 0.652±0.05 | 0.667±0.05 |

**Supplementary Table 25.** Comparison of ssGSEA with alternative methods on NSCLC dataset

| **Method** | **AUROC** | **AUPR** | **F1** | **Accuracy** |
| --- | --- | --- | --- | --- |
| AUCell | 0.639±0.046 | 0.648±0.07 | 0.615±0.035 | 0.614±0.036 |
| UniPath | 0.56±0.042 | 0.602±0.049 | 0.546±0.033 | 0.516±0.035 |
| ssGSEA | 0.718±0.041 | 0.728±0.042 | 0.66±0.043 | 0.653±0.035 |

**Supplementary Table 26.** Comparison of ssGSEA with alternative methods on RCC dataset

| **Method** | **AUROC** | **AUPR** | **F1** | **Accuracy** |
| --- | --- | --- | --- | --- |
| AUCell | 0.564±0.099 | 0.552±0.075 | 0.562±0.093 | 0.562±0.093 |
| UniPath | 0.544±0.109 | 0.582±0.085 | 0.569±0.086 | 0.534±0.095 |
| ssGSEA | 0.709±0.042 | 0.707±0.065 | 0.637±0.097 | 0.669±0.089 |

**Supplementary Table 27.** Comparison of DrugReSC method with and without imputation on melanoma dataset

| **Method** | **AUROC** | **AUPR** | **F1** | **Accuracy** |
| --- | --- | --- | --- | --- |
| DrugReSC with imputation | 0.465±0.076 | 0.467±0.049 | 0.471±0.088 | 0.471±0.088 |
| DrugReSC without imputation | 0.702±0.042 | 0.738±0.039 | 0.652±0.05 | 0.667±0.05 |

**Supplementary Table 28.** Comparison of DrugReSC method with and without imputation on NSCLC dataset

| **Method** | **AUROC** | **AUPR** | **F1** | **Accuracy** |
| --- | --- | --- | --- | --- |
| DrugReSC with imputation | 0.489±0.06 | 0.516±0.05 | 0.479±0.057 | 0.477±0.056 |
| DrugReSC without imputation | 0.718±0.041 | 0.728±0.042 | 0.66±0.043 | 0.653±0.035 |

**Supplementary Table 29.** Comparison of DrugReSC method with and without imputation on RCC dataset

| **Method** | **AUROC** | **AUPR** | **F1** | **Accuracy** |
| --- | --- | --- | --- | --- |
| DrugReSC with imputation | 0.589±0.097 | 0.576±0.079 | 0.556±0.086 | 0.556±0.086 |
| DrugReSC without imputation | 0.709±0.042 | 0.707±0.065 | 0.637±0.097 | 0.669±0.089 |
