# Supplementary figures and images for: DrugReSC: targeting disease-critical cell subpopulations with single-cell transcriptomic data for drug repurposing in cancer
Source: Brief Bioinform. 2024 Sep 30;25(6):bbae490. doi: 10.1093/bib/bbae490 (PMC11442150; doi:10.1093/bib/bbae490)

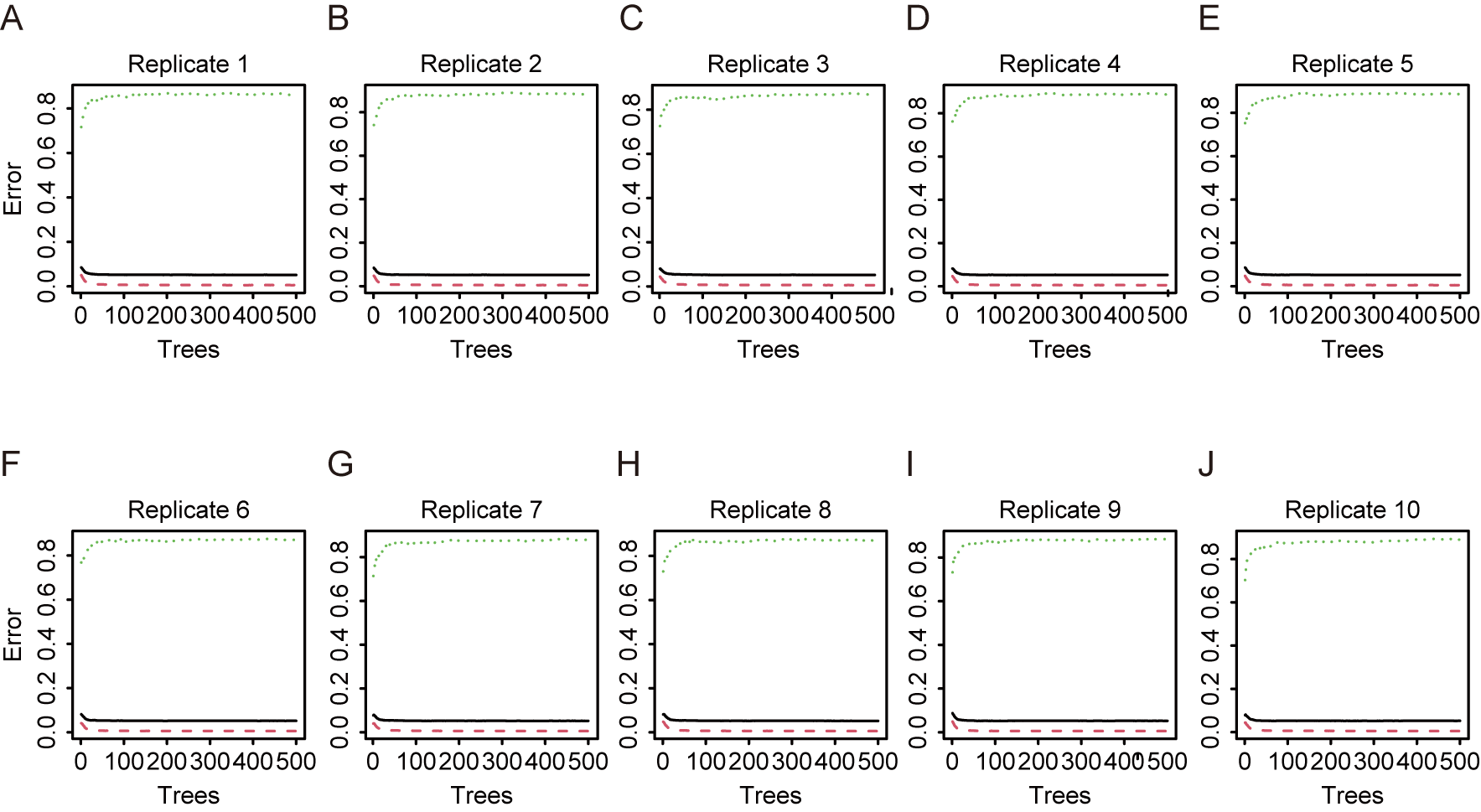

Supplement: Supplementary_bbae490 [file supplementary_bbae490.zip › Supplementary Figure 1. Performance of random forest model with varying numbers of trees in melanoma cases.tif]

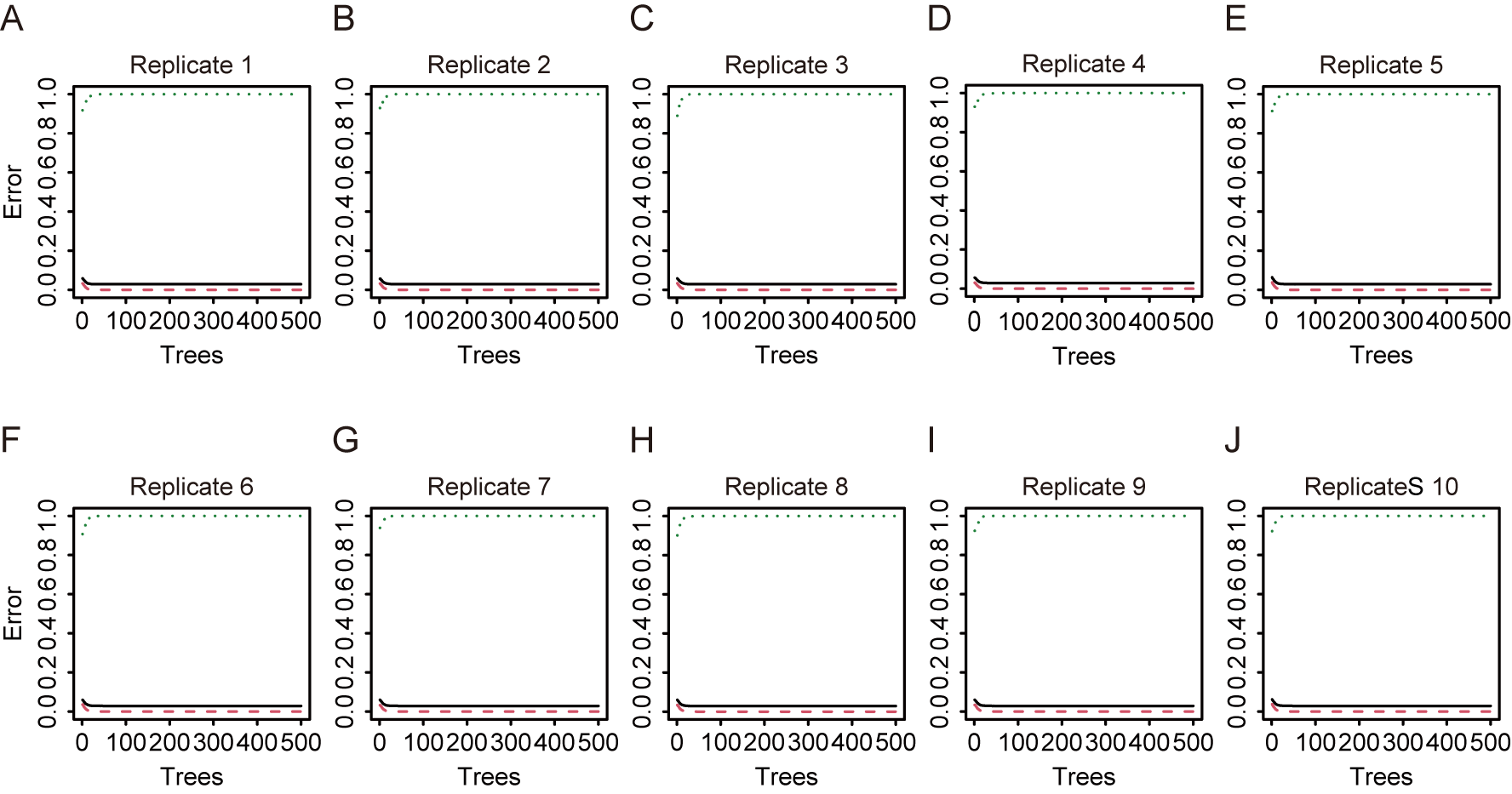

Supplement: Supplementary_bbae490 [file supplementary_bbae490.zip › Supplementary Figure 2. Performance of random forest model with varying numbers of trees in NSCLC cases.tif]

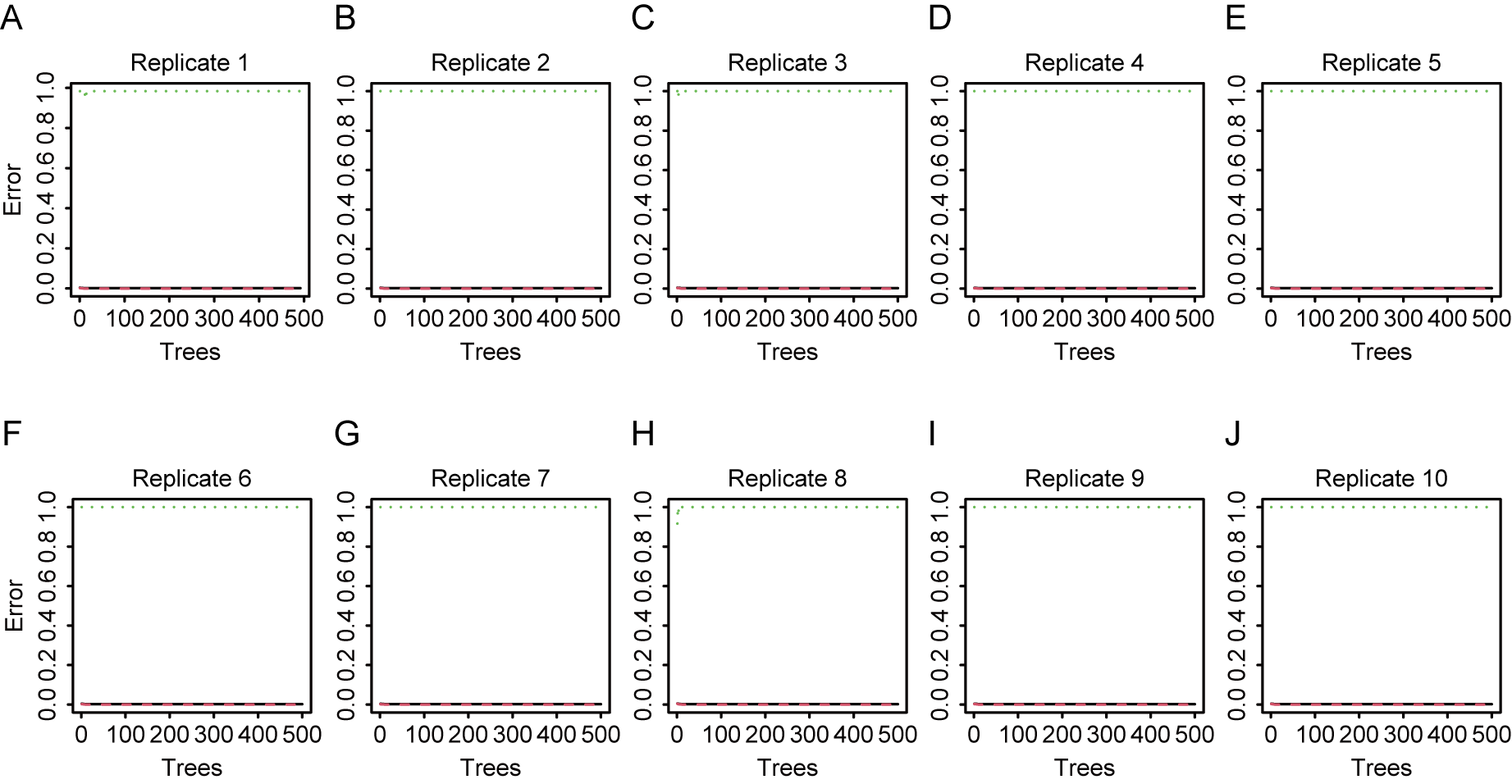

Supplement: Supplementary_bbae490 [file supplementary_bbae490.zip › Supplementary Figure 3. Performance of random forest model with varying numbers of trees in RCC cases.tif]

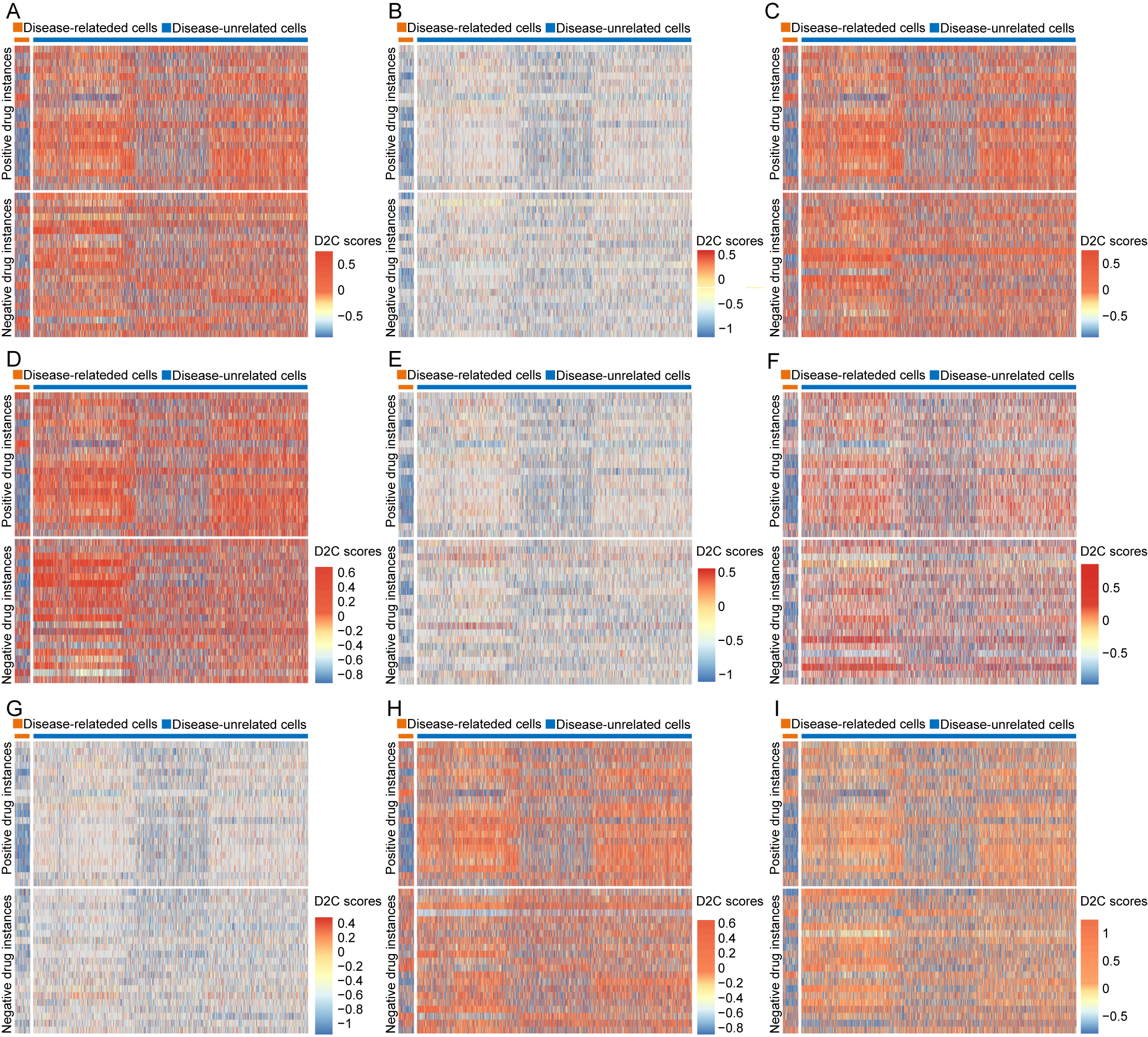

Supplement: Supplementary_bbae490 [file supplementary_bbae490.zip › Supplementary Figure 4. The remaining nine heatmaps of D2C scores computed using DrugReSC of positive and negative drug instances between melanoma-related cells and the other cells.tif]

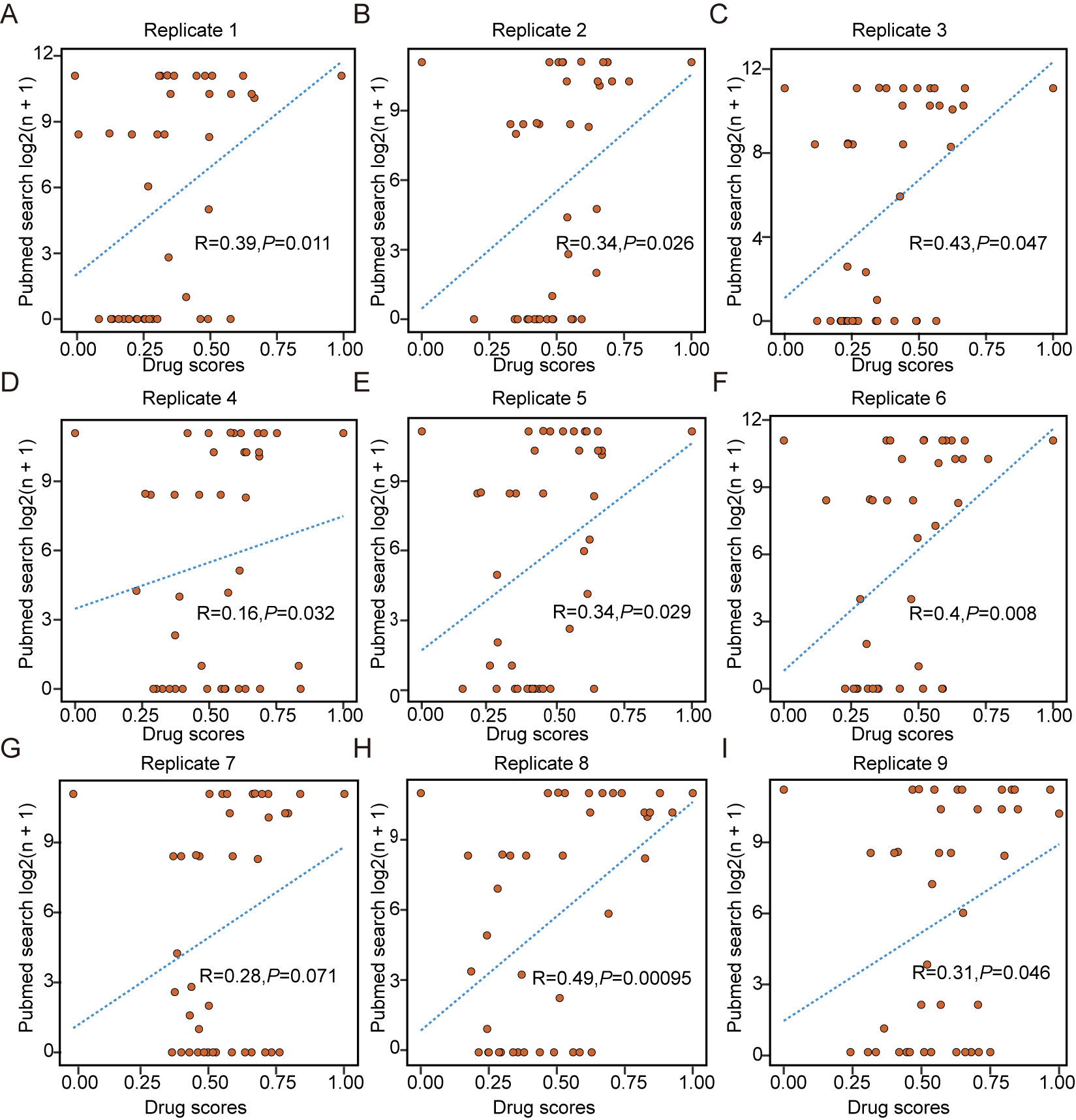

Supplement: Supplementary_bbae490 [file supplementary_bbae490.zip › Supplementary Figure 5. The remaining nine plots depict Pearson correlation between DrugReSC-predicted drug importance scores for melanoma and PubMed search results (log2(n + 1)).tif]

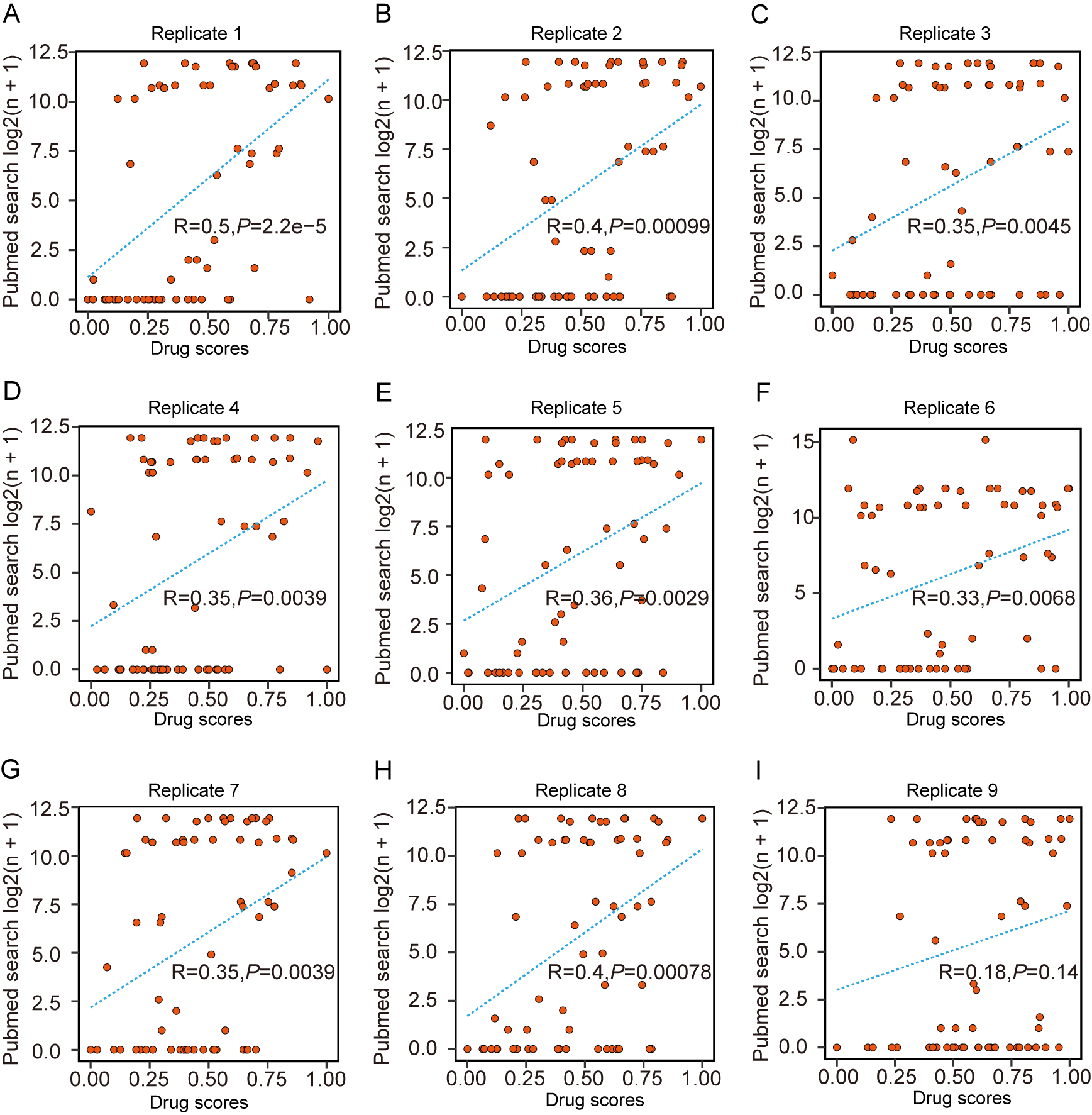

Supplement: Supplementary_bbae490 [file supplementary_bbae490.zip › Supplementary Figure 6. The remaining nine plots depict Pearson correlation between DrugReSC-predicted drug importance scores for NSCLC and PubMed search results (log2(n + 1)).tif]

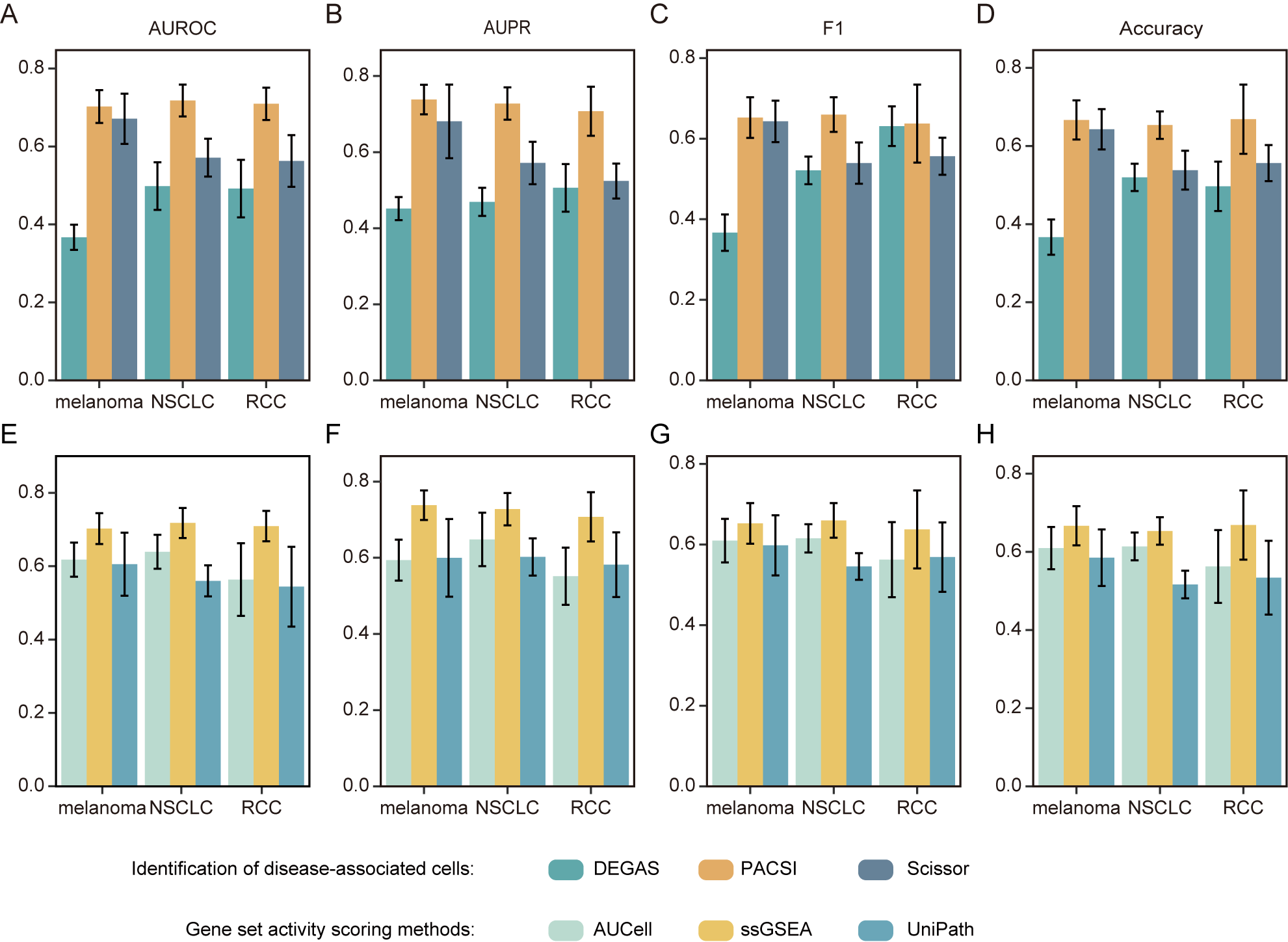

Supplement: Supplementary_bbae490 [file supplementary_bbae490.zip › Supplementary Figure 7. Performance comparison of DrugReSC with alternative methods.tif]

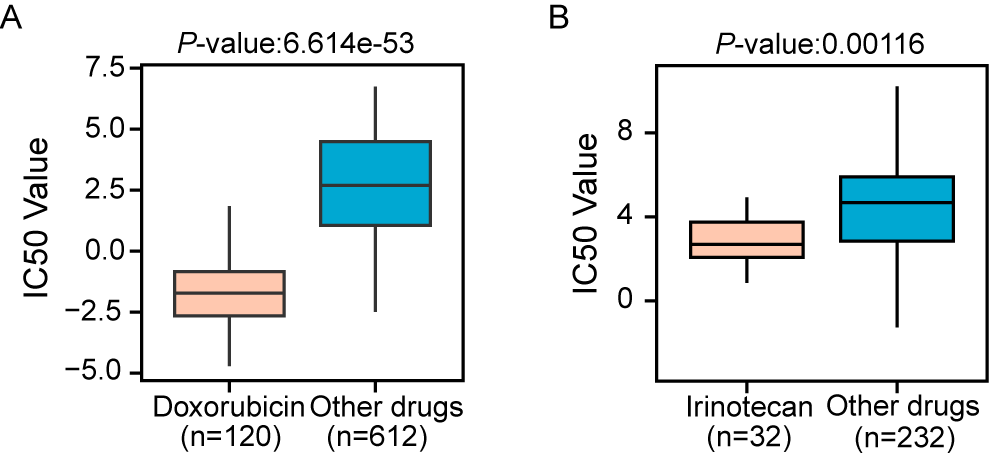

Supplement: Supplementary_bbae490 [file supplementary_bbae490.zip › Supplementary Figure 8. Comparison of activity distribution between DrugReSC-identified candidate drugs and randomly selected drugs.tif]

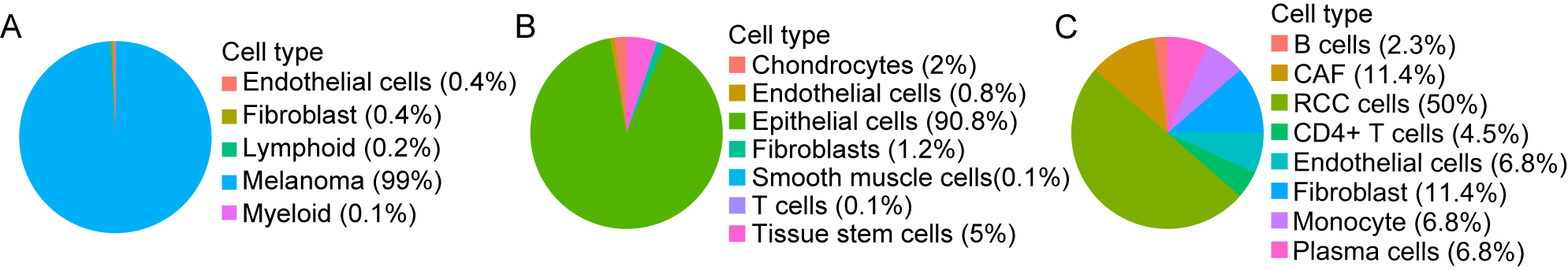

Supplement: Supplementary_bbae490 [file supplementary_bbae490.zip › Supplementary Figure 9. The distribution of PACSI-identified cells by cell types..tif]
